# An LC-QToF MS based method for untargeted metabolomics of human fecal samples

**DOI:** 10.1007/s11306-020-01669-z

**Published:** 2020-04-03

**Authors:** Ken Cheng, Carl Brunius, Rikard Fristedt, Rikard Landberg

**Affiliations:** Division of Food and Nutrition Science, Department of Biology and Biological Engineering, Chalmers University of Technology, 412 96 Gothenburg, Sweden

**Keywords:** Fecal sample, Freeze-drying, Method optimization, Short-chain fatty acids, Untargeted fecal metabolomics, XCMS

## Abstract

**Introduction:**

Consensus in sample preparation for untargeted human fecal metabolomics is lacking.

**Objectives:**

To obtain sample preparation with broad metabolite coverage for high-throughput LC–MS.

**Methods:**

Extraction solvent, solvent ratio and fresh frozen-vs-lyophilized samples were evaluated by metabolite feature quality.

**Results:**

Methanol at 5 mL per g wet feces provided a wide metabolite coverage with optimal balance between signal intensity and saturation for both fresh frozen and lyophilized samples. Lyophilization did not affect SCFA and is recommended because of convenience in normalizing to dry matter.

**Conclusion:**

The suggested sample preparation is simple, efficient and suitable for large-scale human fecal metabolomics.

**Electronic supplementary material:**

The online version of this article (10.1007/s11306-020-01669-z) contains supplementary material, which is available to authorized users.

## Introduction

Untargeted metabolomics aims to comprehensively profile the small molecules in a biological system for different purposes, such as disease prediction, studies of mechanisms and assessment of the exposome (Karu et al. 2018). Establishing robust methods that allow profiling of a wide number of metabolites in large-scale sample sets at high throughput remains a major challenge (Deda et al. 2015, 2018; Matysik et al. 2016).

Research has shown that gut microbiota and their metabolites interact with host metabolism and thereby influence host health (Lamichhane et al. 2018). Feces is an attractive matrix for untargeted profiling that can be easily and non-invasively obtained, and is rich in endogenous host metabolites as well as metabolites derived from gut microbiota and its interaction with xenobiotics, such as bio-transformed food metabolites (Matysik et al. 2016). It was also recently shown that the fecal metabolome to a large extent reflects the gut microbiota composition and activity and it was concluded that fecal metabolomics provides a potential to explore links between microbiome composition and the host phenotypes (Zierer et al. 2018).

LC–MS-based untargeted metabolomics is a sensitive and widely applied analytical tool, whereas so far, the application of LC–MS-based untargeted metabolomics on fecal samples is less common than that of NMR or GC–MS (Cesbron et al. 2017; Deda et al. 2017; Karu et al. 2018). Among existing LC–MS studies (e.g. Cesbron et al. 2017; Deda et al. 2017, 2018; Huang et al. 2013; Jiménez-Girón et al. 2015; Loftfield et al. 2016; Lopez-Bascon et al. 2019; Moosmang et al. 2019; Turroni et al. 2016; Yu et al. 2017; Zhao et al. 2012; Zierer et al. 2018), the procedures for sample preparation are typically simple, but a standardized protocol for human fecal sample handling in order to cover a wide range of metabolites is so far lacking (Deda et al. 2015; Karu et al. 2018). Researchers have for instance used: (1) Different types of fecal materials, e.g. fecal water (Yu et al. 2017), fresh fecal samples (Loftfield et al. 2016), frozen-thawed fecal samples (Deda et al. 2017; Huang et al. 2013; Turroni et al. 2016; Zhao et al. 2012) and lyophilized fecal samples (Zierer et al., 2018) and; (2) Different solvents for fecal metabolites extraction, e.g. MeOH (Huang et al. 2013; Zierer et al. 2018), acetonitrile (ACN) (Deda et al. 2018; Zhao et al. 2012), saline water (Jiménez-Girón et al. 2015), and multiple solvents including H_2_O, MeOH, and chloroform in different ratios (Lopez-Bascon et al. 2019; Moosmang et al. 2019). Various extraction methods have been applied for different analytical purpose, e.g. to increase coverage of specific metabolite classes, such as lipids, amino acids, etc. To the best of our knowledge, only very few articles have reported protocol optimization of human (Moosmang et al. 2019) and animal (Cesbron et al. 2017; Lopez-Bascon et al. 2019) fecal samples adapted for untargeted LC–MS-based metabolomics. In brief, these articles seem to favor frozen over lyophilized fecal material, simpler sample pre-treatment (Cesbron et al. 2017; Moosmang et al. 2019), and extraction using single-phase methanol at a feces-solvent-ratio of 1:3 (*w/v*, Cesbron et al. 2017), water at 1:5 (*w/v*, Moosmang et al. 2019), or methanol/water at 1:25 (*w/v*, Lopez-Bascon et al. 2019). Additionally, a filtration step is usually recommended to reduce the risk of changes in metabolic profile and blockage of chromatography instrumental lines (Karu et al. 2018), but the effects of filtration on metabolites coverage of human fecal samples in LC–MS has not been thoroughly evaluated.

Selection of suitable parameters has been based on observed peak shapes and baseline performance of TIC and Base Peak spectra (Cesbron et al. 2017) or on the number of detected signals or identified metabolites (Lopez-Bascon et al. 2019; Moosmang et al. 2019). However, these methods provide only limited information that is not adequate to check the coverage of metabolites in detail: In TIC and BPC, the assessment of low intensity peaks is not well represented by higher-intensity peaks and apparent peak overlap may be deconvoluted when examining m/z-resolved data. The total number of peaks carries only limited information about peak quality and well-defined metabolite subsets do not necessarily reflect the untargeted coverage of the metabolome. Given these potential pitfalls, it has become increasingly common to investigate the detection of peaks or “features” in LC–MS-based untargeted metabolomics studies, since they cover a wider range of the measurable metabolome. However, to avoid erroneous inference, the total number of features should be balanced against other measures for peak/feature quality (Wang et al. 2019).

Thus, in the current study we aimed to obtain a high-throughput and robust method with broad coverage of the human fecal metabolome from untargeted LC–MS metabolomics. We evaluated in particular extraction solvents (MeOH, ACN and H_2_O), solvent ratio (SR, 1 to 40), fecal materials (fresh frozen and freeze-dried fecal sample, FR, FD) and the effects of filtration. In these evaluations, we have assessed feature quality indirectly, by comparing features derived from an XCMS-based pipeline between different protocols. Selected feature parameters included total number of features, proportion of missing values and total intensities. In addition, given their importance in diet-microbiota-health research, we performed a targeted evaluation of short chain fatty acids between the different protocols.

## Materials and method

### Fecal materials

Fecal samples used in the study were obtained from 10 healthy men and women (5 men and 5 women, Sweden) A self-administered collection kit (EasySampler, GP Medical Devices ApS, Denmark) was used from which a 1–3 g sample was taken to a test tube and frozen at − 20 °C immediately after collection until the sample was brought to − 80 °C storage at the laboratory within 24 h.

Pooled fecal samples were prepared for the selection of solvent ratio (SR; pooled from n = 5 randomly selected individuals) and extraction solvent (pooled from all 10 individuals). For comparisons of metabolic profiles between fresh frozen (FR) and freeze-dried (FD) fecal materials, individual fecal samples were analyzed (n = 10). All samples were analyzed in triplicate, except for the solvent ratio test where only one replicate was analyzed (Table S1).

### Sample preparation

#### Selection of solvent ratio

After thawing at 4 °C for 30 min, frozen fecal samples (200, 150, 60, 30, 30, 30 and 30 mg in wet weight) were lyophilized after weighing and then dissolved in MeOH (200, 300, 300, 300, 600, 900 and 1200 µL) in solvent ratio (SR) 1:1, 1:2, 1:5, 1:10, 1:20,1:30 and 1:40 (w/v). Throughout the manuscript, we refer to these ratios in a simplified format, i.e. SR 1, 2, 5, 10, 20, 30 and 40, respectively.

#### Selection of solvent and solvent ratio

Fresh frozen and lyophilized fecal samples (60 mg wet weight) were dissolved in 300 and 600 µL of either MeOH, Acetonitrile (ACN) or H_2_O in SR 5 and 10.

#### Selection of fecal materials

All freeze-drying was conducted overnight (15 h, HetoDrywimmer, − 55 °C). Lyophilized samples were weighed before and after drying and stored at − 80 °C until analysis. Fresh frozen and lyophilized fecal samples (60 mg in wet weight) were dissolved in 300 µL MeOH to get SR 5, vortexed at 1600 rpm for 5 min, ultra-sonicated for 30 min at room temperature, and vortex again. After centrifugation (18,000×*g* for 15 min at 4 °C), the supernatants were transferred and stored at − 80 °C until analysis. Prior to transferring to LC vials, all samples were centrifuged at 18,000×*g* for 30 min at 4 °C. ACN and MeOH in HPLC–MS grade (VWR Chemicals), and Milli-Q H_2_O which was filtered with LC-Pak Polisher (18.2 MΩ at 25 °C, 3–4 ppb TOC, Merck Millipore) were used.

When testing the differences between filtered and non-filtered samples, 96-well Captiva ND plates (1 mL, Agilent) and 96-well collection plates (0.45 mL, Nunc. Thermo) were applied for filtration.

Blank samples followed by in-house mixtures of external standards were injected at the beginning of each sequence to monitor baseline instrument performance, including mass accuracy and retention time. Quality control samples (QCs) were prepared for each test separately, by combining aliquots from all extracts. QC samples were injected regularly throughout each analytical batch (5 injections at the beginning of run (post external standard mixture) for equilibration, at the end and at every 6th sample in each sequence) to monitor the stability and functionality of the instrumental system and to correct for instrumental drift (Brunius et al. 2016).

### Untargeted HPLC‑MS analysis

Samples were analyzed by UHPLC-QTOF MS using an Agilent 1290 Infinity UHPLC system (Santa Clara, CA) interfaced to a quadrupole-time-of-flight mass spectrometer (Agilent 6520 accurate-mass) equipped with an electrospray ion source (ESI). The separation of compounds was achieved on an Acquity UPLC HSS T3 (C18) column (1.8 µm, 2.1 × 100 mm, Waters, Ireland) at 45 °C. The injection volume was 2 µL. A binary eluent system of water (A) and methanol (B), both containing 0.04% of formic acid was used at a flow rate of 0.4 mL/min. The programmed gradient profile was 0–6 min: 5% to 100% B, 6–10.50 min: 100% B, 10.50–10.51 min: 100% to 5% B, 10.51–15 min: 5% B. The ESI was operated at 3500 V in both positive and negative mode and using nitrogen as nebulizing and drying gas. The gas temperature of the source was 175 °C, gas flow 12 L/min and the nebulizing gas pressure 45 psig. The fragmentor voltage was set at 170 V and skimmer at 65 V. Reference masses (149.02332 and 922.009798 in positive mode, and 112.985587 and 996.000725 in negative mode) were used for mass accuracy checking. Mass spectra were recorded from m/z 50 to m/z 1600. Data were acquired in centroid mode at an acquisition rate of 1.67 spectra/s in the Agilent MassHunter software, and converted to mzML format using MSConvert (ProteoWizard 3.0.18285).

### Targeted GC–MS analysis of short-chain fatty acids

We performed a targeted analysis of the short-chain fatty acids (SCFA: acetic-, propionic-, butyric-, isobutyric-, valeric-, isovaleric- and caproic acid) in fresh frozen and lyophilized fecal samples using a GC–MS method to evaluate their potential loss upon freeze-drying. Briefly, 20 mg fresh frozen fecal samples were weighed into test tubes. Lyophilized fecal samples were obtained by freeze-drying overnight (as described above). Samples were then diluted in 4 mL MilliQ H_2_O and mixed thoroughly with metal beads (2 mm) for 5 min. A portion of 400 µL of the supernatants was mixed with 100 µL meta- phosphoric acid (16% *w/v* in MilliQ H_2_O) containing internal standard (15 nmol acrylic acid, L04280, Alfa Aesar, USA) for 5 min using a vortex. Propyl formate (300 µL) was added to the samples and mixed for 5 min. Samples were centrifuged at 16,000 g 4 °C for 15 min and 150 µL of the upper organic layer was collected in GC vials for analysis.

Samples were analyzed by a Shimadzu GC–MS-TQ8030 (Tokyo, Japan), fast scanning triple quadrupole GC system with a PAL autosampler. The sample (2 µL) was injected in split-less mode and helium (2 mL/min) was used as carrier gas. SCFA were separated on a ZB-FFAP column (30 m, 0.25 µm ID, no. 7HG-G009-11; Phenomenex, USA). The initial oven temperature was set at 40 °C for 1 min, then ramped to 250 °C at a rate of 40 °C/min. The final temperature was held for 2 min giving a total runtime of 8 min per sample. Electron impact ionization (250 °C) mode was used. Selected ion monitoring was performed with one quantification ion and one confirmation ion (m/z), respectively: Acetic acid m/z 60 and 45, propionic acid m/z 74 and 57, isobutyric acid m/z 73 and 57, butyric acid 73 and 60, isovaleric acid m/z 87 and 60, valeric acid m/z 73 and 60, caproic acid m/z 73 and 60 and for the internal standard acrylic acid m/z 72 and 45. Quantification was made based on an 8 point standard curve in ranges normally observed in fecal samples: 10–1280 µM for acetic acid (Honeywell), 4–512 µM for propionic acid (Alfa Aesar) and butyric acid (Sigma-Aldrich) and 0.5–64 µM for the other SCFA (isobutyric acid and valeric acid purchased from Alfa Aesar; isovaleric acid and caproic acid from Sigma-Aldrich). The SCFA concentrations were linearly regressed against the ratio of SCFA/acrylic acid. Samples, standards and blanks were analyzed randomly by the GC–MS system. Integrations were performed with GCMSsolution workstation software (Shimadzu GCMS-TQ series).

### Data pre-processing

The term ‘feature’ refers to a mass spectral peak, i.e. a molecular entity with a unique mass to charge ratio (m/z) and retention time (RT) measured by the LC–MS instrument. Data was deconvoluted using the XCMS R package v 3.4.1 (Smith et al. 2006). Parameters were optimized specifically per experiment from a combination of IPO-assisted (Libiseller et al. 2015) and manual optimization (Table S2). Signal intensities were first corrected for instrument drift using the within-batch correction method in “BatchCorr” (Brunius et al. 2016) (Fig. S1) and then normalized by fecal dry mass. The features with retention time < 660 s and intensity coefficient of variation (CV) in QCs < 30% were kept for further evaluation in the selection of extraction solvents and solvent ratios.

### Statistical analysis

Number of features, percentage of missing values (NA%), total intensities of features and CV of feature intensities were compared between treatments using paired t-test in R v 3.5.1. Extraction solvent and solvent ratio were analyzed separately per fecal material (FR and FD) using solvent, solvent ratio and their interaction as fixed factors; For comparison between FR and FD, fecal material was used as a fixed factor and individual (n = 10) as a random factor.

## Results and discussion

Between 10,000 and 34,000 features were extracted in positive and negative ionization mode using xcms. Of these, 4000–12,000 features were kept for further evaluation after data filtering (Table S3). This large number of features represent a variety of molecular entities, such as monoisotopic metabolite ions, isotopes, adducts, fragments, dimers, trimers, instrument-specific ions or random noise and thus overestimates the number of actual analytes in the sample matrix (Mahieu and Patti 2017). However, grouping of features into pseudospectra is known to create artefacts (Senan et al. 2019). Thus, we have chosen to present the subsequent evaluations on the level of features although many of them likely represent the same analyte although this may induce over-representation bias. Manual inspection showed good performance with reproducible retention times and intensities in BPC with satisfactory baseline separation and peak shapes in the major peaks of QCs, samples and blank samples (Figs. S2a, 2b).

### Selection of solvent ratio and solvents for extraction

Solvent ratios up to 1:25 have previously been reported (Lopez-Bascon et al. 2019). During our early investigations using SR 20, we had observed signal saturation for some features and therefore extended up to SR 40. Signal saturation for these features was, however, not considerably improved at higher SRs. With decreasing SR from 40 to 5, feature numbers and total intensity of features increased, and the proportion of missing values (NA%) decreased in both positive and negative ESI mode (Fig. S3). The SR 1 was not practical for extraction due to a small liquid volume. Samples prepared with SR 2 had a higher total intensity than SR 5, but slightly lower number of features and higher NA% in both positive and negative mode (Fig. S3). Importantly, several signals at SR 2 at 300–450 s retention were saturated, effectively limiting the practical usability of this solvent ratio. Therefore, the SR 5 and 10 were chosen for further selections.

**Fig. 1:**
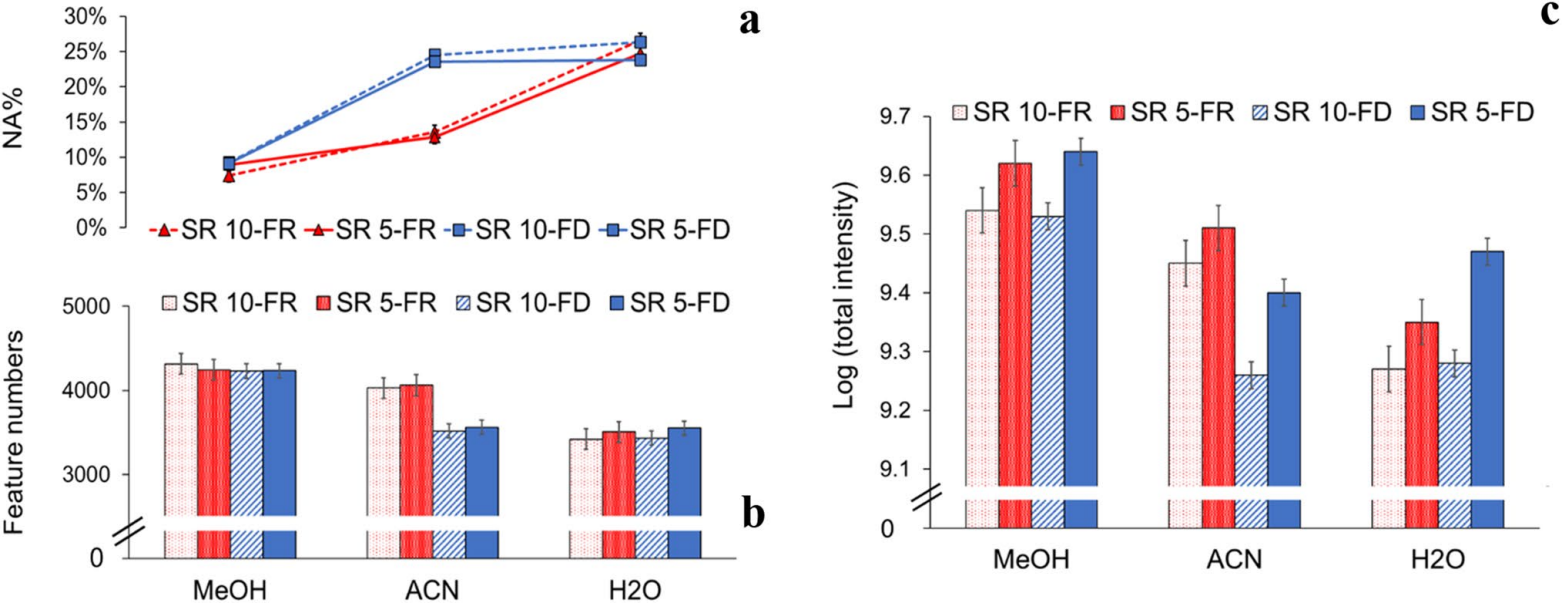
**a** NA%, **b** feature numbers and **c** log (total intensity) in fresh (FR) and lyophilized (FD) fecal samples using MeOH, ACN and H_2_O as extraction solvents at solvent ratio (SR) 10 and 5 (n = 3), results in positive electron spray ionization mode

The choice of solvent affected the number and total intensity of detected features as well as the proportion of missing values in both fresh frozen and lyophilized samples (*P* < 0.0001; Figs. 1, S4). Methanol resulted in a larger number of reproducibly detected features compared to ACN and H_2_O, likely representing a wider coverage of the metabolome. For example, in FD fecal samples at SR 5, 97% of all features detected from any extraction solvent showed up in MeOH, whereas only 86% and 87% of the features were present in ACN and H_2_O, respectively (Fig. S5c). Moreover, the methanol extracts had lower proportion of missing values and higher total intensity than extracts of ACN and H_2_O (Figs. 1, S4). To investigate whether the advantage of methanol as an extraction solvent was related to its use as mobile phases in the chromatographic system, we visually inspected peak shapes. However, we could not observe any obvious differences in peak shape between the extraction solvents, which could be due to the small injection volume. The advantage of methanol as extraction solvent was further supported by Cesbron et al. (2017) and Lopez-Bascon et al. (2019). However, in opposition to these results, Moosmang et al. (2019) instead reported better extraction yield, peak shape and metabolite coverage for water, which they therefore recommend for extraction solvent, at least for reverse phase LC–MS.

Using methanol as extraction solvent also resulted in improved repeatability for sample preparation and linearity within conditions used, compared with MeOH/chloroform (3:1), ACN, ACN/chloroform (3:1) and H_2_O in a targeted GC–MS analysis (Gao et al. 2010). An LC–MS study by Cesbron et al. (2017) found that extraction of fresh frozen feces with methanol produced better peak shape and baseline in total ion and base peak chromatograms, compared with the extraction of lyophilized feces using multiple solvents (MeOH, chloroform and H_2_O). Multi-solvent extraction (MeOH/chloroform/H_2_O) had higher coverage of metabolites in a semi-targeted NMR study, but is more time-consuming, labor intensive, expensive and toxic compared to one-solvent extraction (Karu et al. 2018; Moosmang et al. 2019) and may also result in poorer chromatography (Cesbron et al. 2017), lower MS sensitivity and reproducibility (Karu et al. 2018). Thus, multi-solvent extraction may not be well suited for large-scale sample preparation. As a final note to the selection of extraction solvent, Moosmang et al. (2019) noted that that different solvents extract different metabolite classes. Consequently, one single extraction technique is not sufficient for a holistic overview over the entire fecal metabolome. Thus, even if one particular extraction solvent may have an advantage in terms of general metabolite coverage, other solvents may have advantages for the coverage of specific metabolite classes and the choice of solvent should correspond to the research question at hand as well as the choice of fecal collection method (Wang et al. 2018).

We observed no differences between SR 5 and 10 in feature numbers or NA%, but total intensity was higher at SR 5 (positive ESI mode: *P* = 0.0838 in FR, *P* = 0.0103 in FD; negative ESI mode: *P* = 0.0623 in FR and *P* = 0.0291 in FD), which should facilitate later identification of compounds by MS/MS (Fig. S6). Similarly, Moosmang et al. (2019) reported SR 5 as optimal, since SR 2 was more difficult to process due to limited initial quantity or sample consistency, whereas higher SR 10 led to more diluted extracts.

### Comparison between fresh frozen and lyophilized fecal samples

**Fig. 2:**
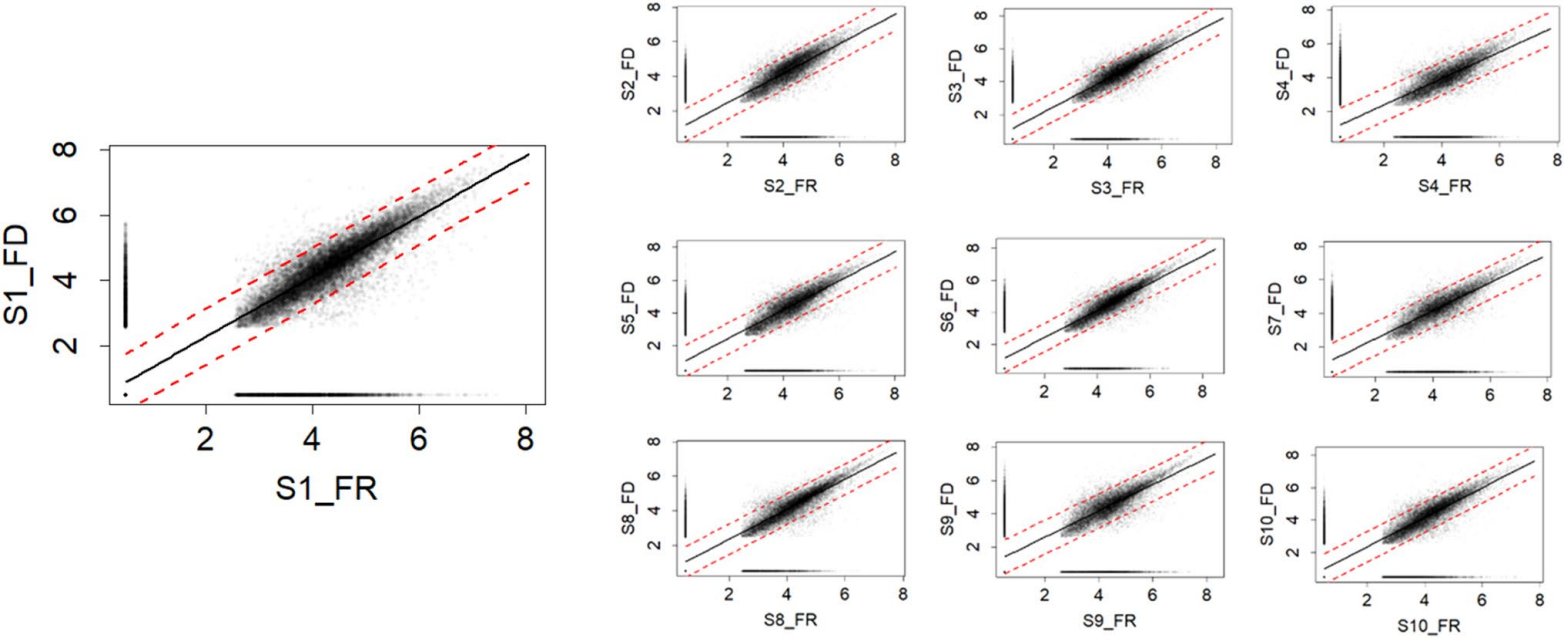
Log-transformed intensity of all features in ten fresh (FR) and lyophilized (FD) fecal sample (S1–S10). All missing values were set to 0.5 before plotting for visual convenience. The red dash lines on the figures are predictive interval lines. Results are in positive electron spray ionization mode

Based on the obtained results, extraction with MeOH at SR 5 was selected for further comparison of fresh frozen vs lyophilized fecal samples. After normalization to dry matter content, features in lyophilized and fresh frozen fecal samples had very similar intensities and proportion of missing values (Figs. 2, S7), although lyophilized fecal samples had slightly higher number of features, lower proportion of missing values and higher total intensity in both positive and negative ESI mode (Table S4). Larger differences in number of detected features were instead observed between individuals (Fig. S8).

Moosmang et al. (2019) also reported similar number of features between lyophilized and fresh frozen fecal samples, indicating that the choice of method does not affect metabolite coverage appreciably. On the other hand, they also reported large discrepancies in signal intensities between lyophilized and fresh frozen fecal samples and hypothesized that these discrepancies may be related to improved extraction efficacy for the lyophilized material. Our results, on the other hand, indicate that the observed discrepancies may instead be related to dry matter content and care should be taken to normalize samples to dry matter.

To further investigate systematic occurrence of missing values in the data, we compared features exclusively missing in FD and FR: 95% and 96% of exclusive missing features in FD and FR samples were present in ≦ 3 samples in both positive and negative modes, indicating that systematic missingness according to preparation (FR vs FD) was only a minor issue. In fact, only 7 and 9 of all the 12,517 analyzed features were exclusively missing from all FD and FR samples, respectively (Table S4).

Whether to use fresh frozen or lyophilized samples for fecal metabolomics is a matter of debate, since each procedure may provide advantages and disadvantages. Freeze-drying has been recommended (Karu et al. 2018; Ng et al. 2012), because difference in water content in fecal samples could have a substantial impact on the analytical outcomes not only due to the influence on weight measurement, but also due to alterations in the lipophilicity of the extraction system. However, some researchers (Deda et al. 2018) proposed that the lyophilization process may increase the risk of losing volatile compounds. However, given the similarity in intensities between fresh and lyophilized samples in the present study, this seems unlikely. SCFA comprise important volatile metabolites derived from gut microbial fermentation of dietary fiber and their potential health effects beyond their established role as fuel for the gut epithelium has received much attention lately (Koh et al. 2016). We consequently performed a targeted comparison of lyophilized vs fresh frozen samples with regards to loss of SCFA. In our study, SCFA were extracted and measured from fresh frozen and lyophilized fecal samples in parallel. We found no observable difference in their content (Table S6). This observation is supported by the biophysical properties of the fatty acids. The pKa of SCFA range between 4.76 for acetic acid to 4.88 for caproic acid (Osuka et al. 2012). The average fecal pH for a healthy person is around 6.6, leaving the SCFA deprotonated which may limit the evaporation of SCFA in feces (Osuka et al. 2012). As expected, when acidifying lyophilized fecal samples, lower contents of SCFAs were detected compared with fresh frozen samples, suggesting evaporation when pH is getting closer to the pKa-values of the acids (data not shown). The impact of freeze-drying on other volatile metabolites in fecal samples has not been specifically investigated in this study.

Given the similarity in feature quality between fresh frozen and lyophilized fecal samples, with a slight advantage for lyophilized samples, freeze-drying allows the additional advantage to normalize to dry matter, without decreasing overall metabolite coverage, which helps to overcome the issue with the large variability in water content in feces, such as fecal samples from diarrhea and constipation patients. Additionally, it has been reported that working with dried fecal samples is less laborious, especially for grinding (Moosmang et al. 2019), more reproducible and also prevents bacterial growth (Karu et al. 2018).

Moreover, no differences were observed between filtered and non-filtered samples regarding chromatogram baseline, number of features or proportion of missing values in our study. Filtered samples did, however, have higher total intensity of all features than non-filtered (Fig. S9 and Table S7). However, without filtration, we observed successively increasing back pressure, retention time deviations and ultimately LC failure, most likely from deposition at the inlet of column. Our method development therefore suggested that the filtration step should be added to improve analytical robustness in large-scale analyses. This approach was also suggested in a recent review (Karu et al. 2018).

There are limitations in our protocol. In the sample preparation, the already high intrinsic sample heterogeneity in the fecal sample matrix could have been exacerbated by weighing in different amounts for the different solvent ratios. However, this seems to be of minor given the associations of feature parameters with solvent ratio (Fig. S3). There may also be differences in precipitation and extraction efficiency in relation to freeze-drying for specific metabolites, however systematic effects on the metabolome coverage seem minor (Fig. 2). In addition, in our effort to simplify the protocol, we only considered pure unmixed solvents and observed that almost all of the features detected in water and ACN were in fact also detected using methanol. However, the investigation of simple one-phase solvent mixtures, especially water:MeOH, could have contributed additional important information, e.g. on signal intensities as a proxy for extraction efficiency and recovery.

Importantly, to make a full, in-depth assessment of method performance and validation such as ion suppression, recovery, extraction efficiency, solvent saturation and limit of quantification, more direct and targeted methods than the ones we have employed are needed. However, a comprehensive, direct analysis of a sufficiently large representative portion of the metabolome required to extend results into generalizations valid for the coverage of the untargeted metabolome is far beyond the scope of this work. We instead chose an indirect methodology consisting of proxy assessment of feature quality, which still represents improvement beyond assessing only the total number of features, visual inspection of base peak chromatogram including peak shape of a sub-selection of major peaks. Another limitation in the study design was the omission of procedural blanks, which would have permitted accurate assessment of contaminant peaks. However, a crude analysis of features that did not differ in intensity between SR 5 and 10 suggested that potential artefacts (from e.g. contaminants and instrumentation) and features outside the linear range was consistent between the investigated extraction solvents and that these may constitute up to approximately 12% of the measured features (Fig S10, Table S3). Consequently, the feature quality parameters that we employed, albeit highly informative, could consequently be biased as metrics of the feature coverage and quality.

In conclusion, we found that MeOH was superior to ACN and H_2_O for fecal extraction to obtain a wide coverage of reproducible, robust metabolite features with high intensity. A solvent ratio of 5 mL solvent per g feces (wet weight) provided an optimal balance between signal intensity and instrument saturation. Fresh frozen and lyophilized fecal material showed similar results with regard to number and intensity of features as well as proportion of missing values and content of volatile SCFA. Considering the highly variable water content in fecal samples, we therefore suggest using lyophilized fecal samples as a convenient approach to normalize metabolite feature intensities to dry matter.

## Electronic supplementary material

Below is the link to the electronic supplementary material.

Supplementary file1 (DOCX 2481 kb)

## References

[CR1] Brunius C, Shi L, Landberg R (2016). Large-scale untargeted LC-MS metabolomics data correction using between-batch feature alignment and cluster-based within-batch signal intensity drift correction. Metabolomics.

[CR2] Cesbron N, Royer AL, Guitton Y, Sydor A, Le Bizec B, Dervilly-Pinel G (2017). Optimization of fecal sample preparation for untargeted LC-HRMS based metabolomics. Metabolomics.

[CR3] Deda O, Chatziioannou AC, Fasoula S, Palachanis D, Raikos Ν, Theodoridis GA, Gika HG (2017). Sample preparation optimization in fecal metabolic profiling. Journal of Chromatography B: Analytical Technologies in the Biomedical and Life Sciences.

[CR4] Deda O, Gika HG, Theodoridis GA, Theodoridis GA, Gika HG, Wilson ID (2018). Rat fecal metabolomics-based analysis. Metabolic profling: Methods and protocols, methods in molecular biology.

[CR5] Deda O, Gika HG, Wilson ID, Theodoridis GA (2015). An overview of fecal sample preparation for global metabolic profiling. Journal of Pharmaceutical and Biomedical Analysis.

[CR6] Gao X, Pujos-Guillot E, Sébédio J-L (2010). Development of a quantitative metabolomic approach to study clinical human fecal water metabolome based on trimethylsilylation derivatization and GC/MS analysis. Analytical Chemistry.

[CR7] Huang H, Zhang A, Cao H, Lu H, Wang B, Xie Q (2013). Metabolomic analyses of faeces reveals malabsorption in cirrhotic patients. Digestive and Liver Disease.

[CR8] Jiménez-Girón A, Ibáñez C, Cifuentes A, Simó C, Muñoz-González I, Martín-Álvarez PJ (2015). Faecal metabolomic fingerprint after moderate consumption of red wine by healthy subjects. Journal of Proteome Research.

[CR9] Karu N, Deng L, Slae M, Guo AC, Sajed T, Huynh H (2018). A review on human fecal metabolomics: Methods, applications and the human fecal metabolome database. Analytica Chimica Acta.

[CR10] Koh A, De Vadder F, Kovatcheva-Datchary P, Bäckhed F (2016). From dietary fiber to host physiology: Short-chain fatty acids as key bacterial metabolites. Cell.

[CR11] Lamichhane S, Sen P, Dickens AM, Orešič M, Bertram HC (2018). Gut metabolome meets microbiome: A methodological perspective to understand the relationship between host and microbe. Methods.

[CR12] Libiseller G, Dvorzak M, Kleb U, Gander E, Eisenberg T, Madeo F (2015). IPO: A tool for automated optimization of XCMS parameters. Melliand International.

[CR13] Loftfield E, Vogtmann E, Sampson JN, Moore SC, Nelson H, Knight R (2016). Comparison of collection methods for fecal samples for discovery metabolomics in epidemiologic studies. Cancer Epidemiology Biomarkers and Prevention.

[CR14] Lopez-Bascon MA, Calderon-Santiago M, Arguello H, Morera L, Garrido JJ, Priego-Capote F (2019). Comprehensive analysis of pig feces metabolome by chromatographic techniques coupled to mass spectrometry in high resolution mode: Influence of sample preparation on the identification coverage. Talanta.

[CR15] Mahieu NG, Patti GJ (2017). Systems-level annotation of a metabolomics data set reduces 25 000 features to fewer than 1000 unique metabolites. Analytical Chemistry.

[CR16] Matysik S, Le Roy CI, Liebisch G, Claus SP (2016). Metabolomics of fecal samples: A practical consideration. Trends in Food Science and Technology.

[CR17] Moosmang S, Pitscheider M, Sturm S, Seger C, Tilg H, Halabalaki M, Stuppner H (2019). Metabolomic analysis—Addressing NMR and LC-MS related problems in human feces sample preparation. Clinica Chimica Acta.

[CR18] Ng JSY, Ryan U, Trengove RD, Maker GL (2012). Development of an untargeted metabolomics method for the analysis of human faecal samples using Cryptosporidium-infected samples. Molecular & Biochemical Parasitology.

[CR19] Osuka A, Shimizu K, Ogura H, Tasaki O, Hamasaki T, Asahara T (2012). Prognostic impact of fecal pH in critically ill patients. Critical Care.

[CR20] Senan O, Aguilar-Mogas A, Navarro M, Capellades J, Noon L, Burks D (2019). CliqueMS: A computational tool for annotating in-source metabolite ions from LC-MS untargeted metabolomics data based on a coelution similarity network. Bioinformatics.

[CR21] Smith CA, Want EJ, O’Maille G, Abagyan R, Siuzdak G (2006). XCMS: Processing mass spectrometry data for metabolite profiling using nonlinear peak alignment, matching and identification. Analytical Chemistry.

[CR22] Turroni S, Fiori J, Rampelli S, Schnorr SL, Consolandi C, Barone M (2016). Fecal metabolome of the Hadza hunter-gatherers: A host-microbiome integrative view. Scientific Reports.

[CR23] Wang L, Naser FJ, Spalding HL, Patti GJ, Fendt S-M, Lunt SY (2019). A protocol to compare methods for untargeted metabolomics. Metabolic signalling: Methods and protocols, methods in molecular biology.

[CR24] Wang Z, Zolnik CP, Qiu Y, Usyk M, Wang T, Strickler HD (2018). Comparison of fecal collection methods for microbiome and metabolomics studies. Frontiers in Cellular and Infection Microbiology.

[CR25] Yu M, Jia H, Zhou C, Yang Y, Zhao Y, Yang M, Zou Z (2017). Variations in gut microbiota and fecal metabolic phenotype associated with depression by 16S rRNA gene sequencing and LC/MS-based metabolomics. Journal of Pharmaceutical and Biomedical Analysis.

[CR26] Zhao YY, Cheng XL, Wei F, Bai X, Lin RC (2012). Application of faecal metabonomics on an experimental model of tubulointerstitial fibrosis by ultra performance liquid chromatography/high-sensitivity mass spectrometry with MS^E^ data collection technique. Biomarkers.

[CR27] Zierer J, Long T, Telenti A, Spector T, Menni C (2018). The fecal metabolome as a functional readout of the gut microbiome. Nature Genetics.

