# Supplementary material for: An LC-QToF MS based method for untargeted metabolomics of human fecal samples
Source: Metabolomics. 2020 Apr 3;16(4):46. doi: 10.1007/s11306-020-01669-z (PMC7125068; doi:10.1007/s11306-020-01669-z)
Supplement: Supplementary file 1 — Supplementary file1 (DOCX 2481 kb) [file 11306_2020_1669_MOESM1_ESM.docx]

**Supplementary materials (*Metabolomics*)**

**An LC-QToF MS based method for untargeted metabolomics of human fecal samples**

Ken Cheng, Carl Brunius, Rikard Fristedt, Rikard Landberg

Division of Food and Nutrition Science, Department of Biology and Biological Engineering, Chalmers University of Technology, SE-412 96 Gothenburg, Sweden

Corresponding authors: Tel.: +46 (0)31 772 2732. Email: [rikard.landberg@chalmers.se](mailto:rikard.landberg@chalmers.se) (ORCID: 0000-0002-8525-4194) and [cheng-ken@live.cn](mailto:cheng-ken@live.cn) (ORCID: 0000-0003-3910-0220).

**Supplementary Tables**

Table S1 Experimental design in tests.

| Tests | SR_test | Solvent _test | Fecal material_test | |
| --- | --- | --- | --- | --- |
| Parameter | SR | Solvent and SR | FD and FR |  |
| Sample | Pooled sample from 5 individuals | Pooled sample from 10 individuals | 10 individuals |  |
| Fecal material | FD | FR and FD | FR and FD |  |
| Replicate | 1 | 3 | 3 |  |
| Treatment | SR_1  SR_2  SR_5  SR_10  SR_20  SR_30  SR_40 | MeOH+SR_5+FR  ACN+SR_5+FR  H_2_O+SR_5+FR  MeOH+SR_10+FR  ACN+SR_10+FR  H_2_O+SR_10+FR  MeOH+SR_5+FD  ACN+SR_5+FD  H_2_O+SR_5+FD  MeOH+SR_10+FD  ACN+SR_10+FD  H_2_O+SR_10+FD | FR  FD |  |
| Solvent | MeOH | MeOH  ACN  H_2_O | MeOH |  |

ACN acetonitrile; FD freeze-dried fecal materials; FR fresh fecal materials; MeOH methanol; SR solvent ratio.

Table S2 Parameters of XCMS in positive (+) and negative (-) electrospray ionization mode used for feature extraction and alignment

|  | SR_test+ | Solvent_test+ | Fecal material_test+ | SR_test- | Solvent_test- | Fecal material_test- | |
| --- | --- | --- | --- | --- | --- | --- | --- |
| *Centwave for peak picking* | | | | | | |  |
| peakwidth | c(6.64, 44.4) | c(6.12, 47.5) | c(6.12, 57.5) | c(3, 75) | c(3, 72.5) | c(3, 55) | |
| mzdiff | -0.001 | 0.0012 | 0.00208 | 0.00065 | 0.00505 | -0.00265 | |
| *Obiwarp for alignment* | | | | | | |  |
| response | 14.86 | 1 | 1 | 15.4 | 10 | 10 | |
| gapInit | 0.568 | 0.4 | 0.24 | 0.64 | 0.34 | 0.3 | |
| gapExtend | 2.4 | 2.4 | 2.7 | 2.46 | 2.4 | 2.7 | |
| *Density for corresponde* | | | | | | |  |
| bw | 1 | 1 | 1 | 1 | 1 | 1 | |
| binsize | 0.003 | 0.035 | 0.035 | 0.0003 | 0.025 | 0.015 | |

Following parameters were the same for all tests: ppm 15, noise 500, snthresh 10, prefilter c(3, 1000), binsize for obiwarp 1, minfrac 0.75.

Table S3. Numbers of features extracted using xcms and after data filtration* in each experiment.

|  | Before data filtering | | After data filtering | |
| --- | --- | --- | --- | --- |
|  | Positive | Negative | Positive | Negative |
| SR_test | 34044 | 22409 | 12698 | 8832 |
| Solvent_test | 12812 | 9688 | 6151 | 6308 |
| Fecal material_test | 23464 | 25456 | 3823 | 8694 |

* data filtration includes removing features with retention time larger than 660 seconds, and CV of QC samples larger than 30%.

Table S4 Feature number, NA% and log (total intensity) in fresh and freeze-dried samples (n=5), results in positive and negative electron spray ionization mode.

|  | Freeze-dried | Fresh | *P* values |
| --- | --- | --- | --- |
| Positive electron spray ionization mode | | | |
| Feature number | 13874 ± 562.9 | 13614 ± 611.1 | 0.062 |
| NA% | 38% ± 0.03 | 39% ± 0.03 | 0.062 |
| Log(total intensity) | 9.58 ± 0.14 | 9.51 ± 0.14 | 0.015 |
| Negative electron spray ionization mode | | | |
| Feature number | 14829 ± 722.7 | 14485 ± 806.7 | 0.008 |
| NA% | 40% ± 0.03 | 42% ± 0.03 | 0.008 |
| Log(total intensity) | 9.40 ± 0.12 | 9.38 ± 0.12 | 0.091 |

Table S5 Exclusive missing features in FD and FR samples (n=10) in positive (+) and negative (-) electron spray ionization mode.

|  | mzmed | Rtmed | mzmin | mzmax | rtmed (s) | rtmin | rtmax |
| --- | --- | --- | --- | --- | --- | --- | --- |
| Exclusive missing features in FD | | | | | | | |
| FT03138 (+) | 243.24 | 7.22 | 243.22 | 243.24 | 432.91 | 431.23 | 433.60 |
| FT04612 (+) | 299.28 | 7.64 | 299.27 | 299.29 | 458.68 | 457.83 | 459.89 |
| FT11178 (+) | 511.76 | 2.92 | 511.74 | 511.77 | 175.06 | 175.06 | 175.65 |
| FT13905 (+) | 593.55 | 7.64 | 593.54 | 593.55 | 458.62 | 457.83 | 459.89 |
| FT00224 (-) | 110.99 | 0.69 | 110.98 | 110.99 | 41.43 | 40.88 | 42.07 |
| FT07723 (-) | 409.24 | 7.99 | 409.24 | 409.24 | 479.29 | 478.08 | 479.29 |
| FT21323 (-) | 849.51 | 6.48 | 849.50 | 849.51 | 388.79 | 388.17 | 389.26 |
| Exclusive missing features in FR | | | | | | | |
| FT14403 (+) | 608.05 | 5.86 | 608.03 | 608.05 | 351.33 | 349.57 | 352.54 |
| FT02514 (-) | 252.15 | 6.42 | 252.15 | 252.15 | 384.95 | 384.11 | 385.19 |
| FT09145 (-) | 451.45 | 7.73 | 451.45 | 451.45 | 463.81 | 463.20 | 464.82 |
| FT10300 (-) | 479.48 | 10.02 | 479.48 | 479.48 | 601.39 | 600.79 | 601.98 |
| FT17965 (-) | 707.69 | 8.49 | 707.69 | 707.69 | 509.39 | 508.62 | 509.87 |
| FT19013 (-) | 744.63 | 9.63 | 744.62 | 744.63 | 578.00 | 577.10 | 578.85 |
| FT19588 (-) | 769.72 | 8.03 | 769.72 | 769.72 | 482.09 | 481.15 | 482.83 |
| FT19940 (-) | 784.70 | 9.24 | 784.70 | 784.70 | 554.59 | 553.64 | 555.53 |
| FT21847 (-) | 875.70 | 10.72 | 875.70 | 875.70 | 643.08 | 642.48 | 644.27 |

Table S6 SCFAs content in fresh and lyophilized fecal samples. Results represents mean values from one individual and two quality control samples consisting of mixtures from several individuals, measured in duplicates. The concentrations are in µmol/g.

| SCFAs | Acetic  Acid | Propionic  Acid | Butyric  Acid | IsoButyric  Acid | IsoValeric  Acid | Valeric  Acid | Caproic  Acid |
| --- | --- | --- | --- | --- | --- | --- | --- |
| Fresh | 77.6 ± 12 | 26.1 ± 4.9 | 18.5 ± 3.8 | 1.3 ± 0.5 | 1.8 ± 1.1 | 1.6 ± 0.5 | 0.9 ± 0.2 |
| Lyophilized | 79.3 ± 6.0 | 26.2 ± 0.8 | 18.1 ± 1.1 | 1.2 ± 0.4 | 1.7 ± 0.8 | 1.5 ± 0.4 | 1.1 ± 0.2 |

Table S7 Feature number, NA% and log (total intensity) in filtered and non-filtered samples (n=5), results in positive electron spray ionization mode.

|  | Filtered | Non-filtered | *P* values |
| --- | --- | --- | --- |
| Feature number | 23329 ± 98.1 | 23791 ± 96.7 | 0.056 |
| NA% | 4.5% ± 0.004 | 4.6% ± 0.004 | 0.056 |
| Log(total intensity) | 9.71 ± 0.010 | 9.64 ± 0.018 | 0.015 |

**Supplementary Figures**

**b**

**a**

| **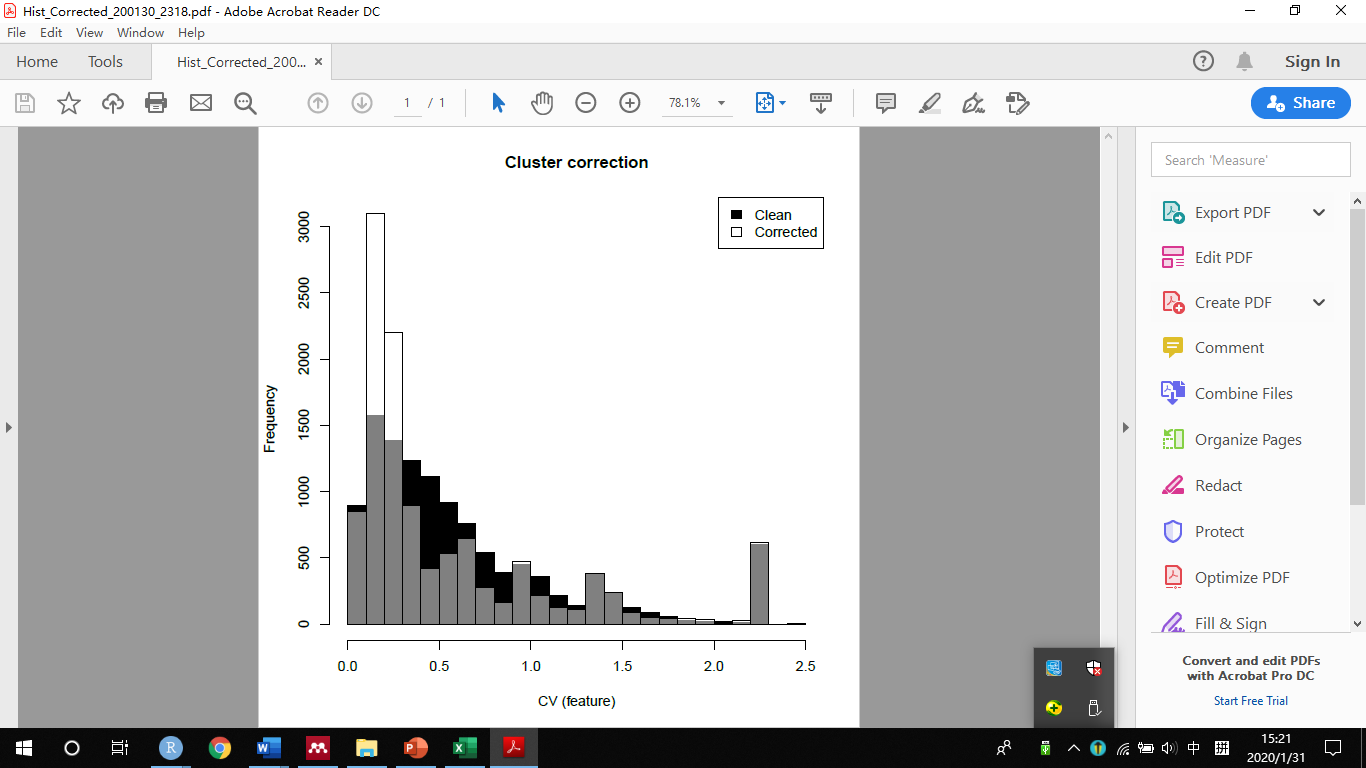**  **c** | **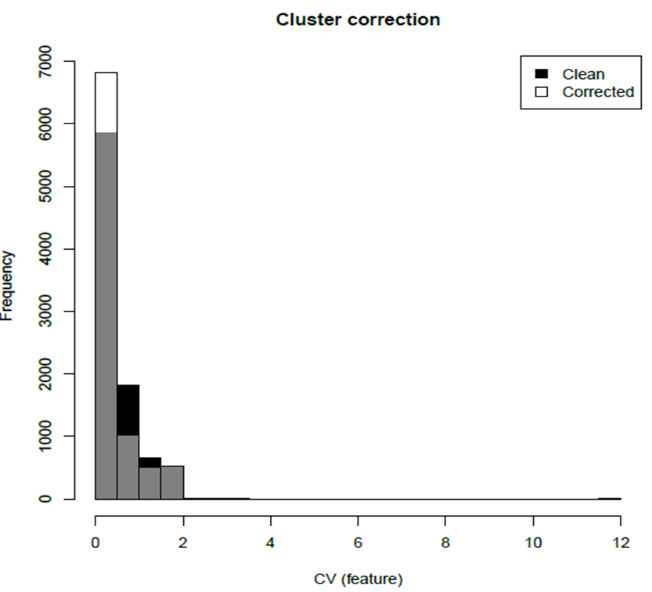**  **d** |
| --- | --- |
| **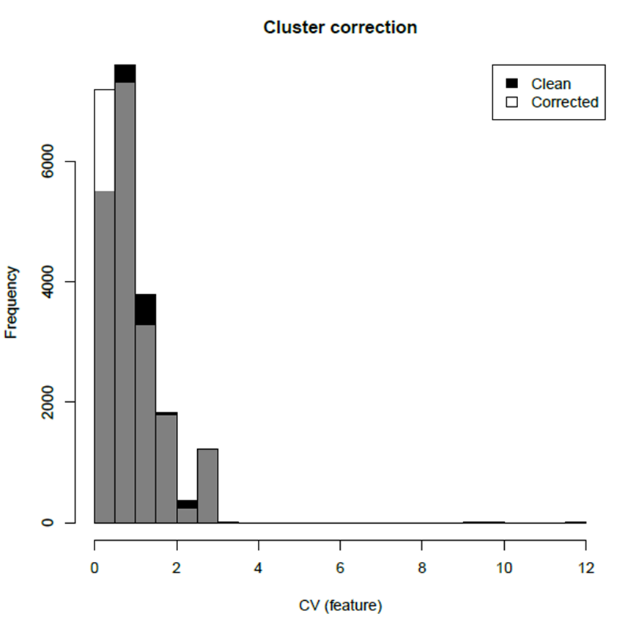** | **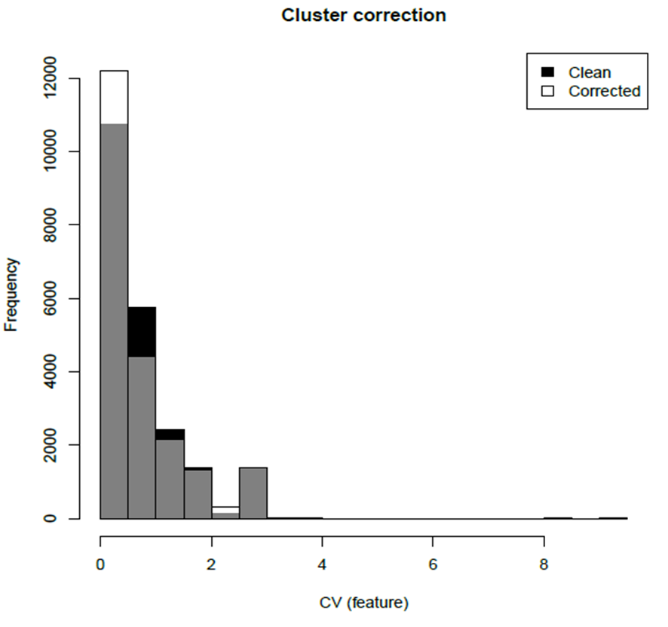** |

Fig. S1 Feature CV (%) distribution of QCs before (clean) and after (corrected) within-batch correction in the solvent_test (a and b in positive and negative ionization mode, respectively) and fecal materials_test (c and d in positive and negative ionization mode, respectively). Plots were obtained from “Batchcorr” R package.

|  | BPC in full RT | BPC in RT 150-250s | BPC in RT 250-350s |
| --- | --- | --- | --- |
| SR_test+ | 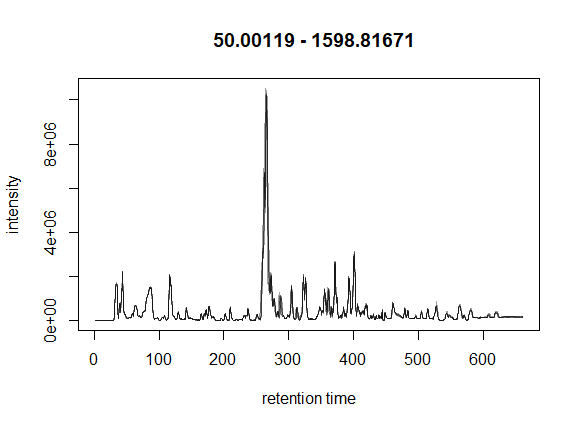 | 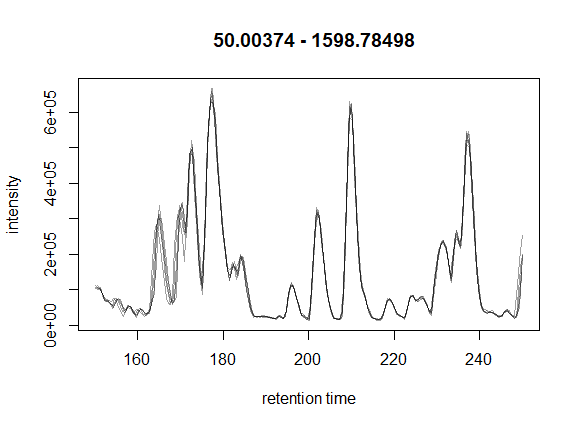 | 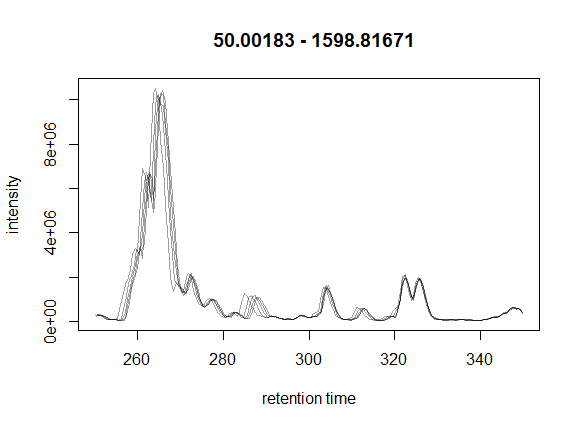 |
| Solvent_test + | 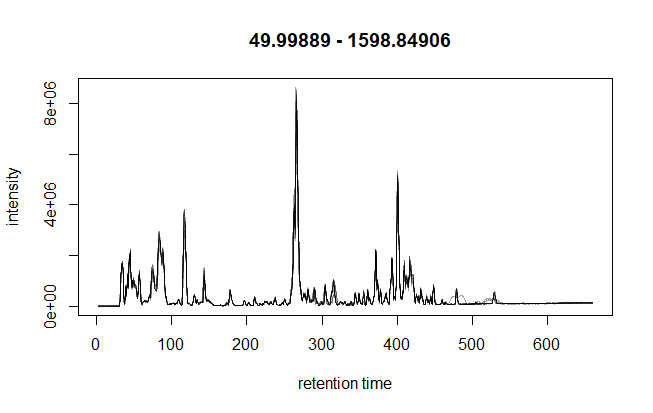 | 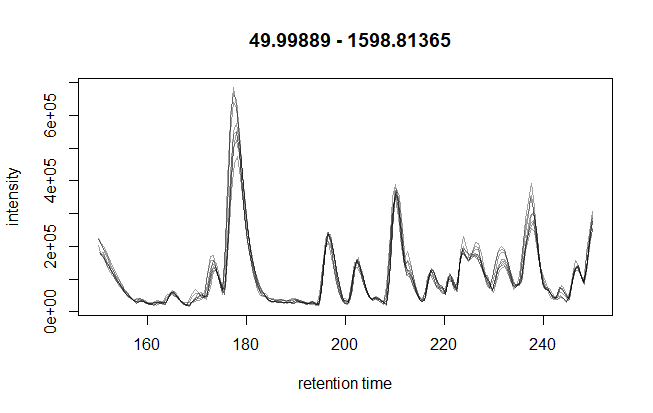 | 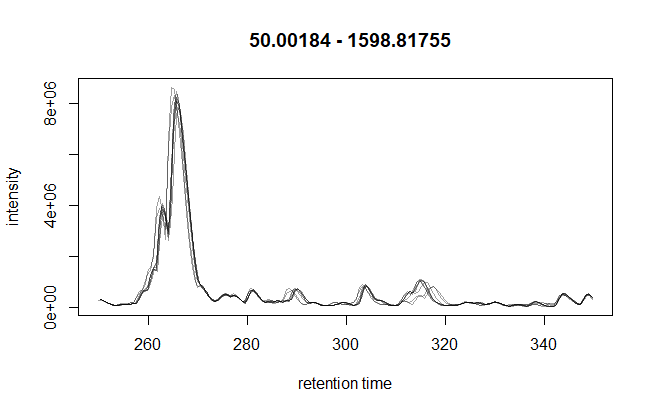 |
| Fecal materials_test + | 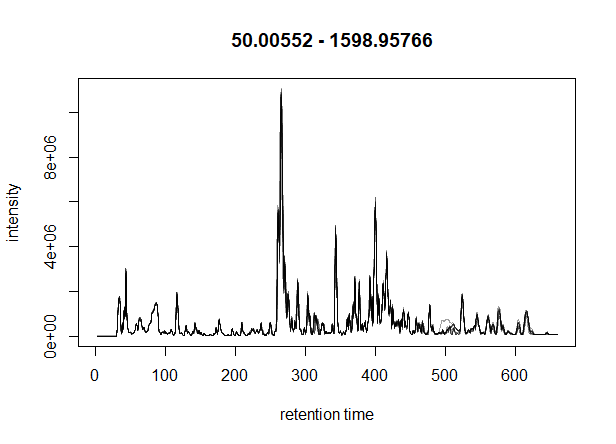 | 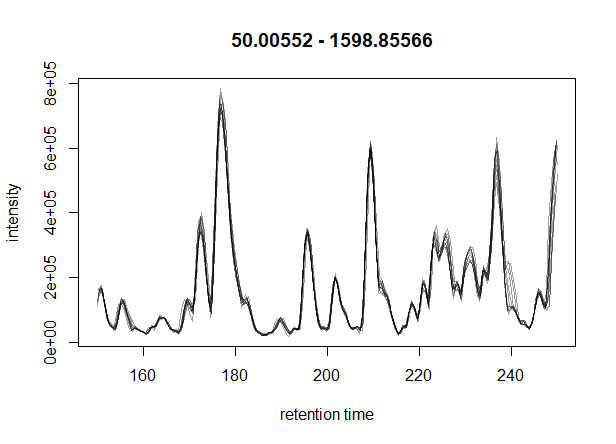 | 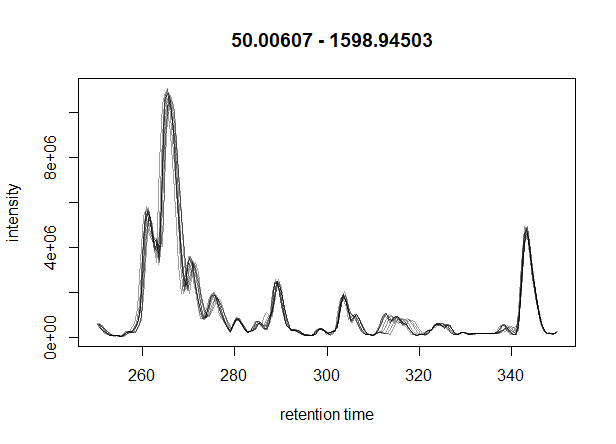 |
| SR_test- | 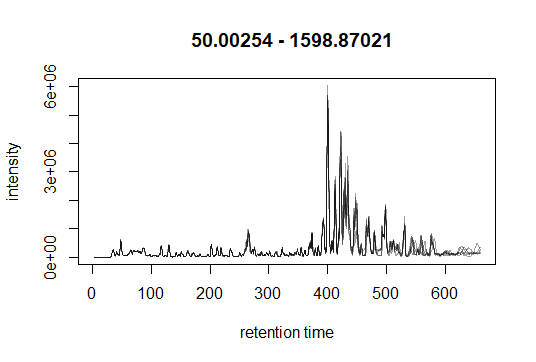 | 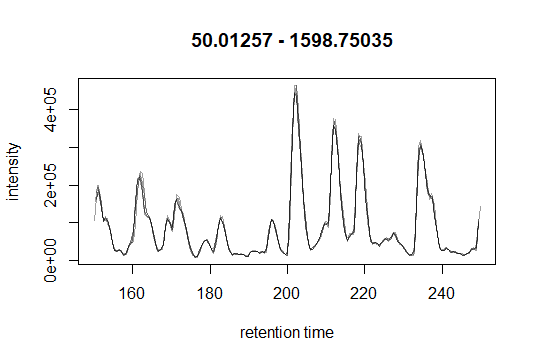 | 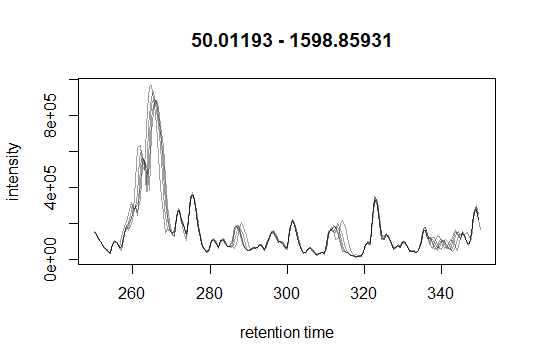 |
| Solvent_test - | 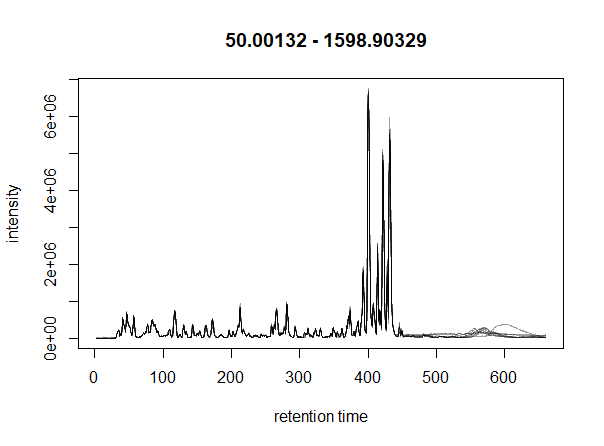 | 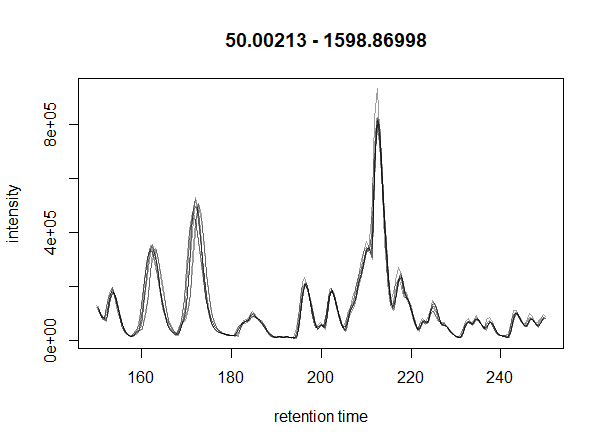 | 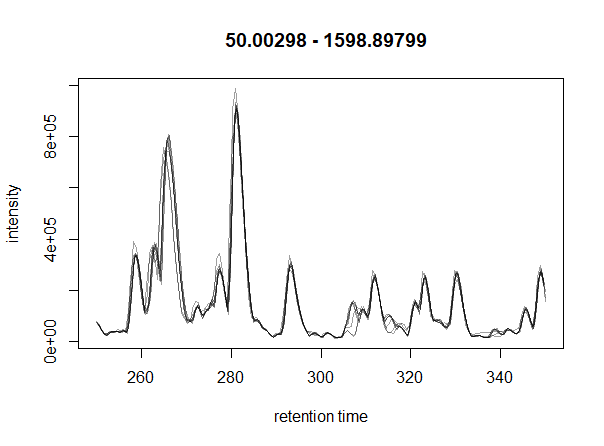 |
| Fecal materials_test - | 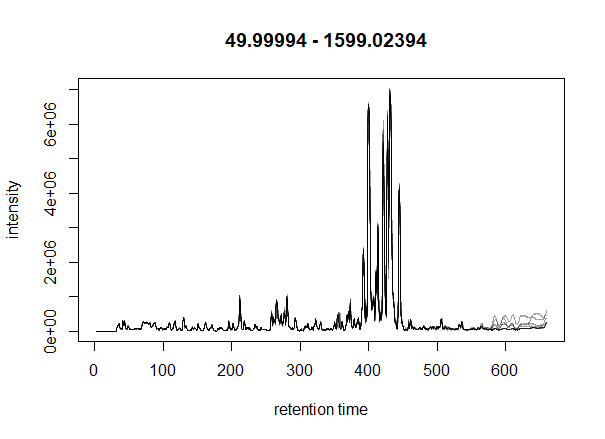 | 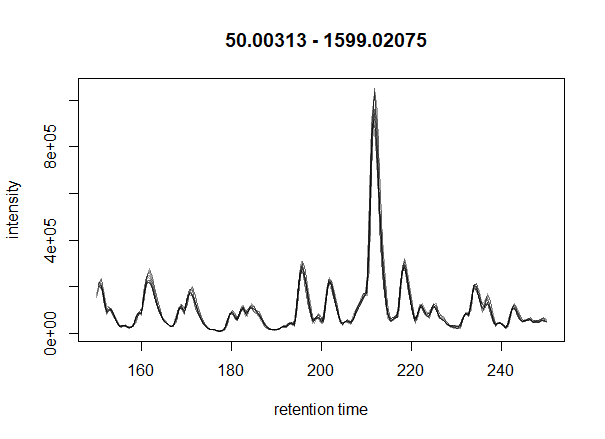 | 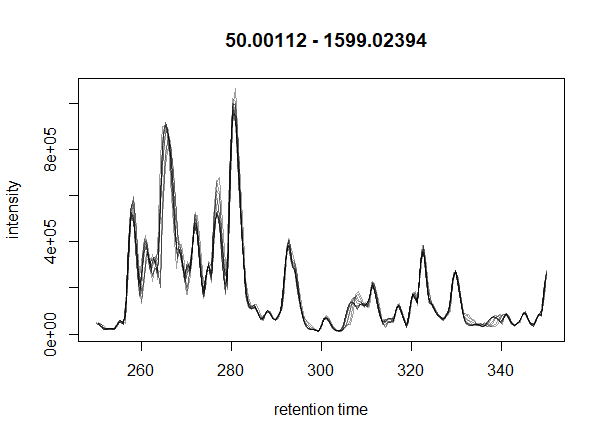 |

Fig. S2a BPC plots of QCs (n=6, 8, and 10 in SR_test, solvent_test and fecal materials_test, respectively) in different retention time (RT) regions (RT 1-600s, 150-250s, 250-350s) in positive (+) and negative (-) ionization mode of different tests prior to retention time adjustment. Similarities in RT and peak intensities between QCs per test suggest a stability of instrument and good performance in peak shape, baseline and peak separation.


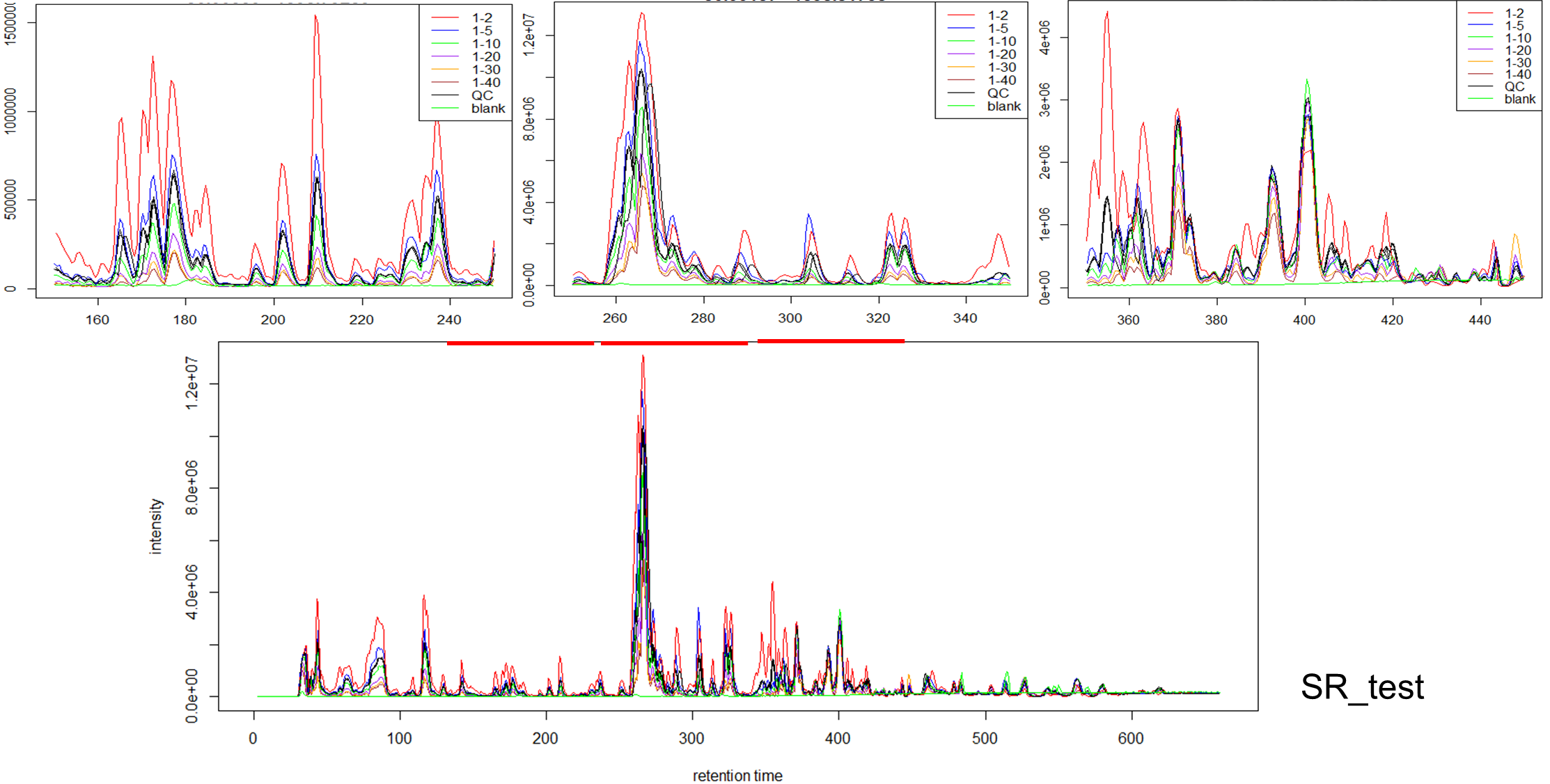


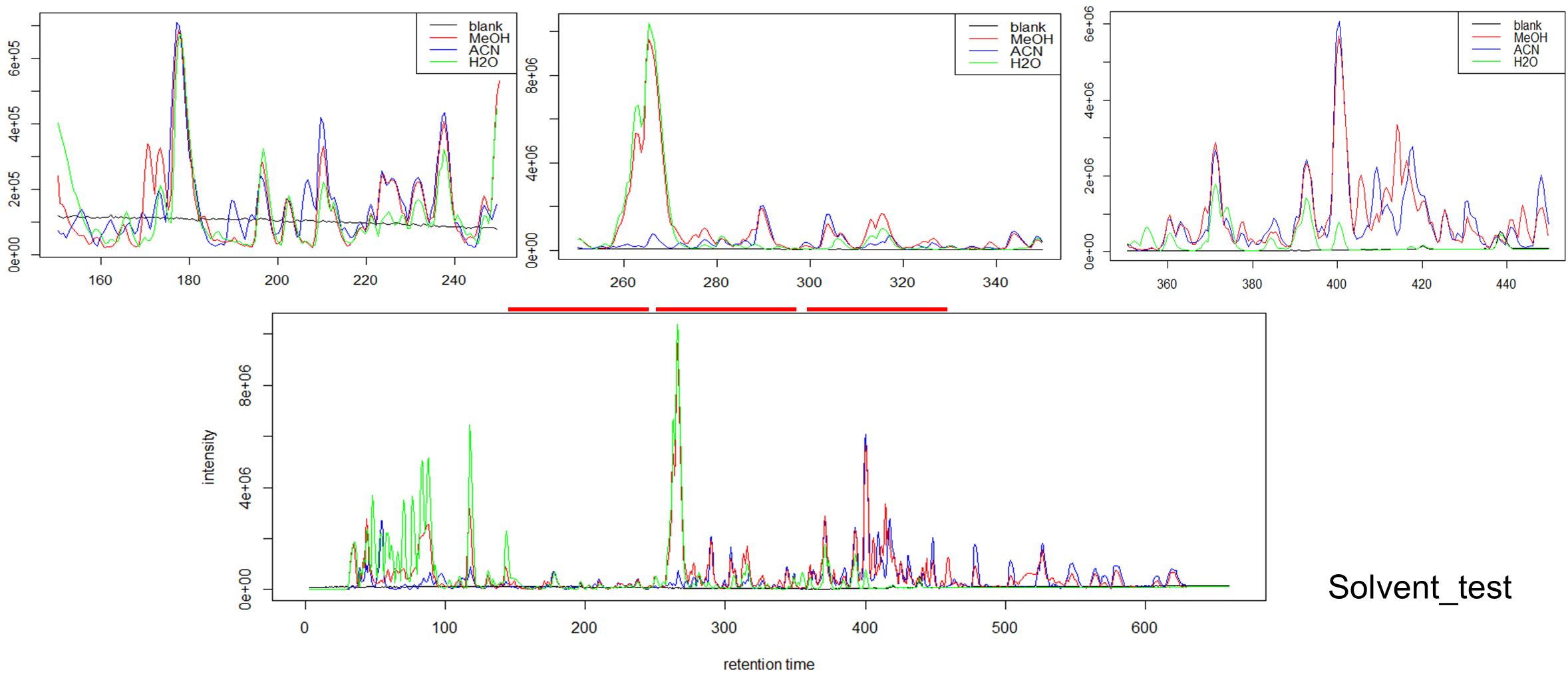


Fig. S2b BPC plots of samples, QCs and blanks in different retention time (RT) regions (RT 1-600s, 150-250s, 250-350s) in the tests of solvent ratio (SR) and solvents. Performance of blanks suggest cleanness of system. The figure of SR_test shows changes in feature intensities in samples extracted with MeOH at different SR (2, 5, 10, 20, 30, 40); The figure of Solvent_test shows that different metabolites were extracted by MeOH, ACN, and H2O.

**b**

**a**

| 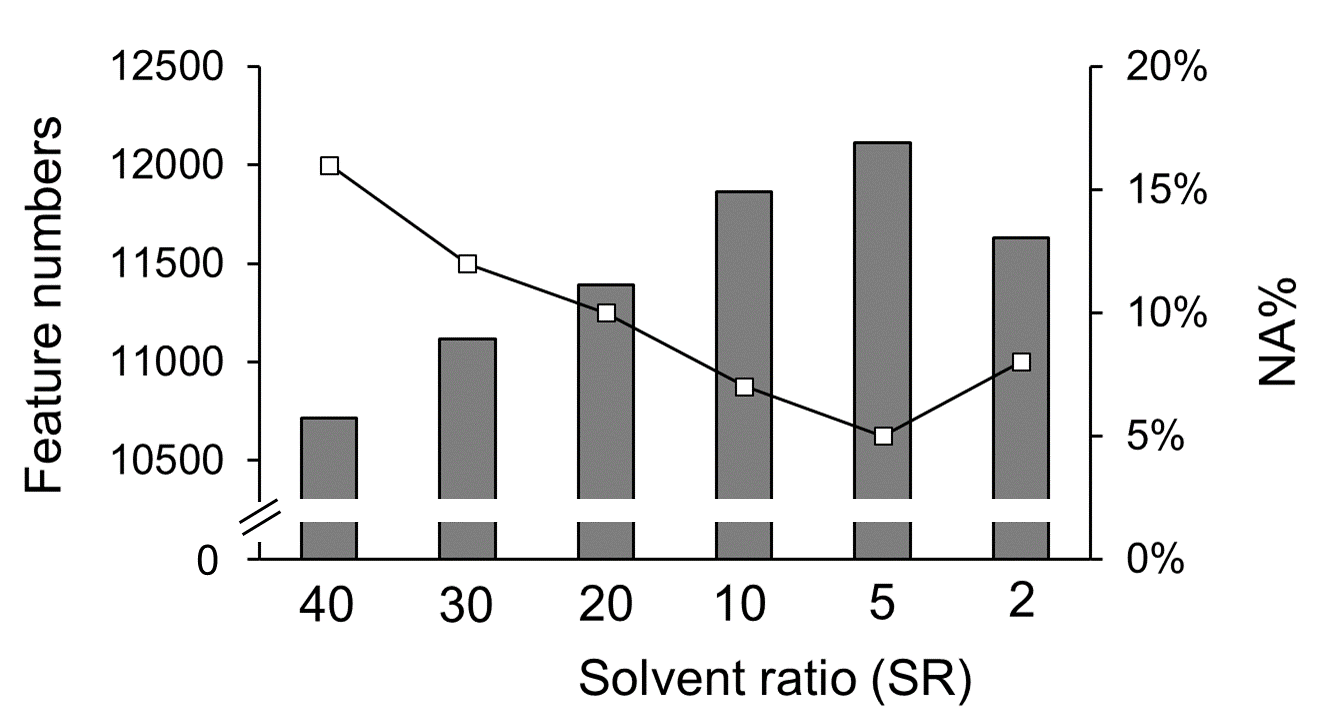  **c** | 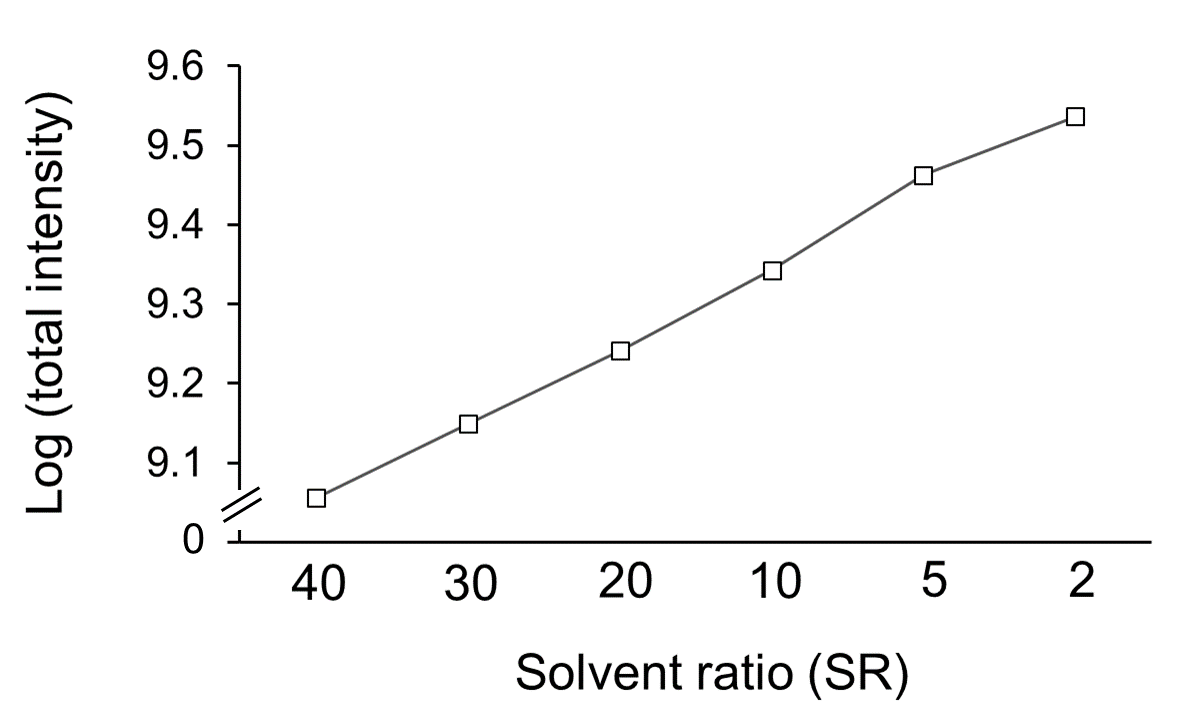  **d** |
| --- | --- |
| 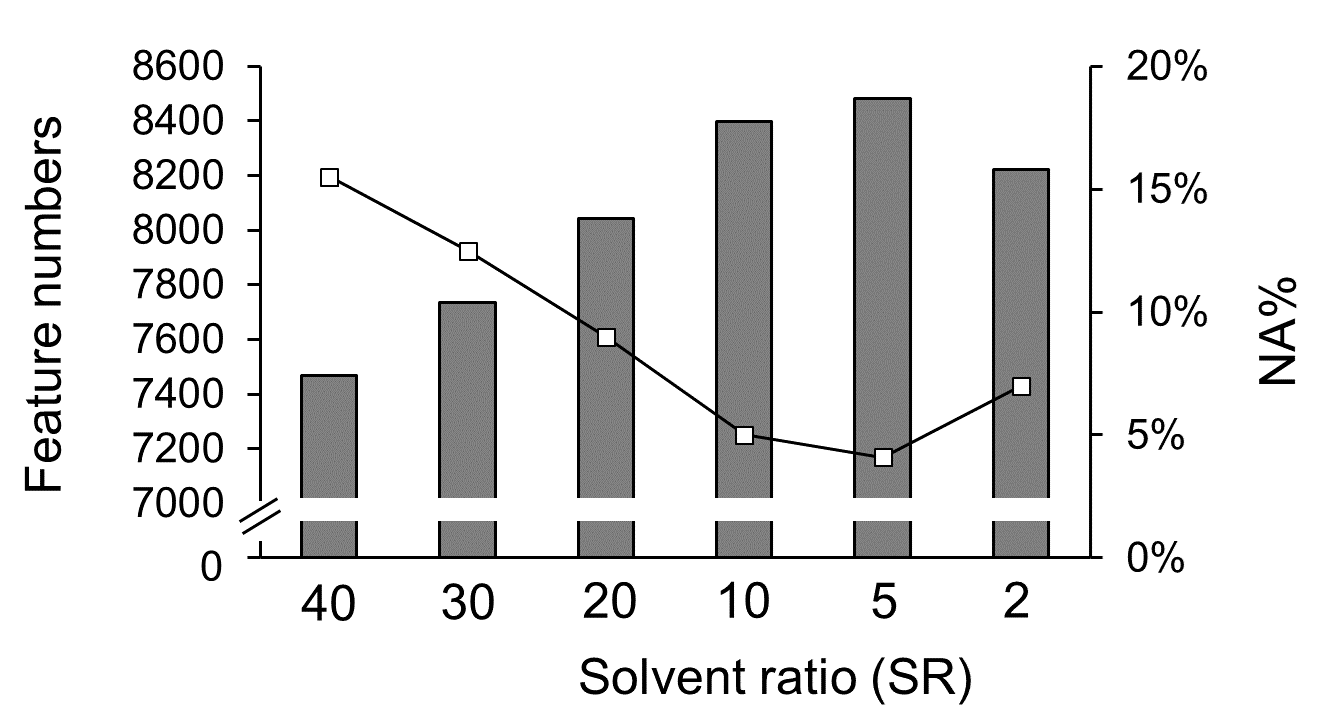 | 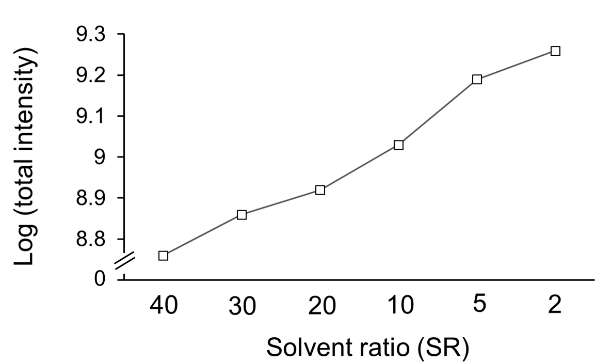 |

Fig. S3 Feature numbers (column graph; a and c), NA% (line graph; a and c) and log(total intensity) (b and d) of fecal extracts using MeOH as extraction solvent at solvent ratio (SR) 40 to 2, results in positive (a and b) and negative (c and d) electron spray ionization mode.

**c**

**a**

| **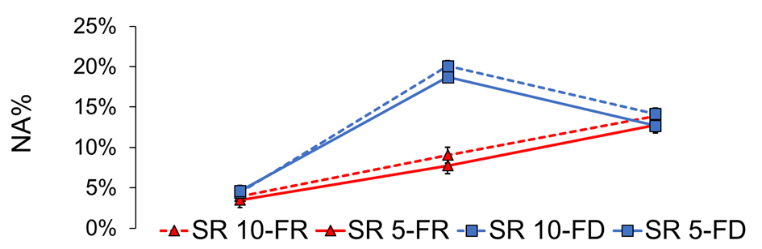** | **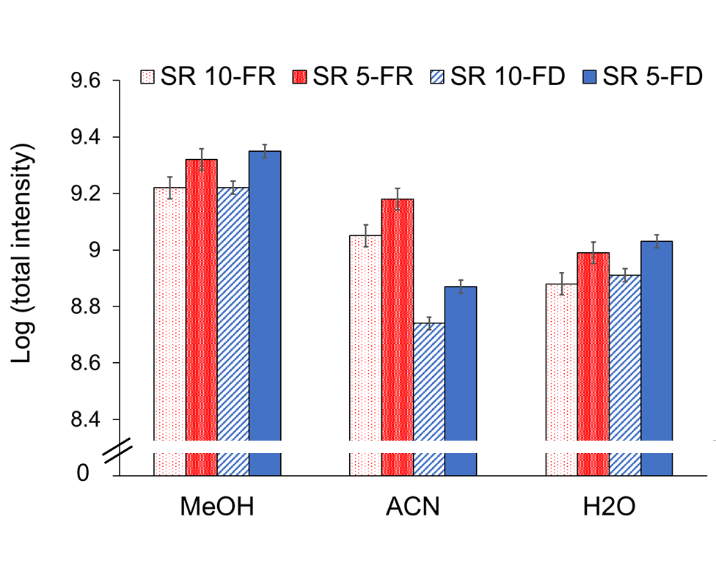** |
| --- | --- |
| **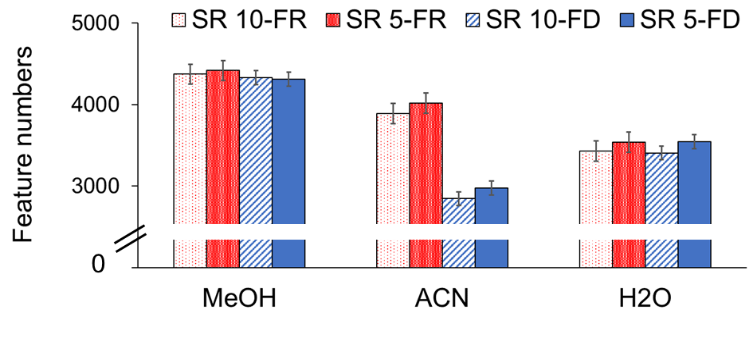**  **b** |  |

Fig. S4 (a) NA%, (b) feature numbers and (c) log (total intensity) of fresh (FR) and freeze-dried (FD) fecal extracts using MeOH, ACN and H_2_O as extraction solvents at solvent ratio (SR) 10 and 5 (n=3), results in negative electron spray ionization mode.

| 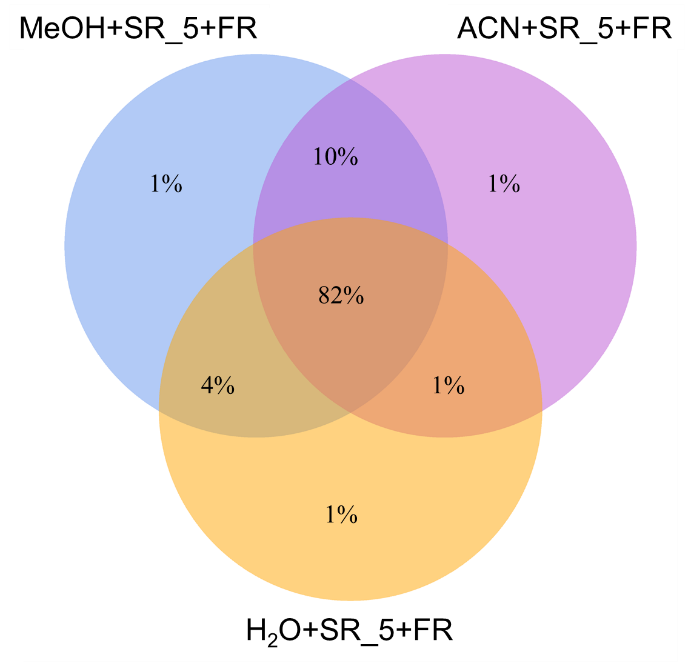 | 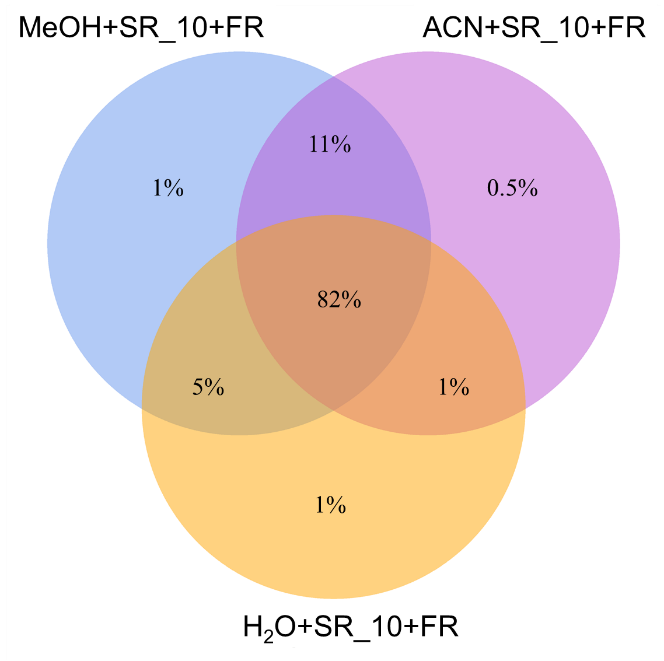 |
| --- | --- |
| 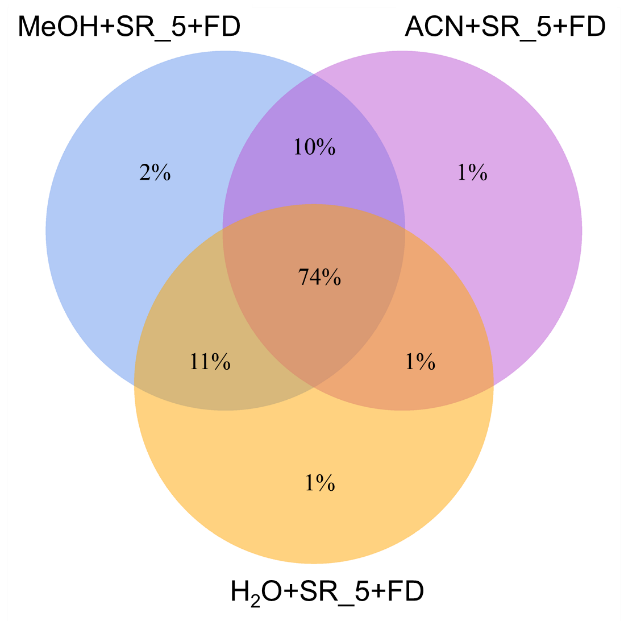  **c** | 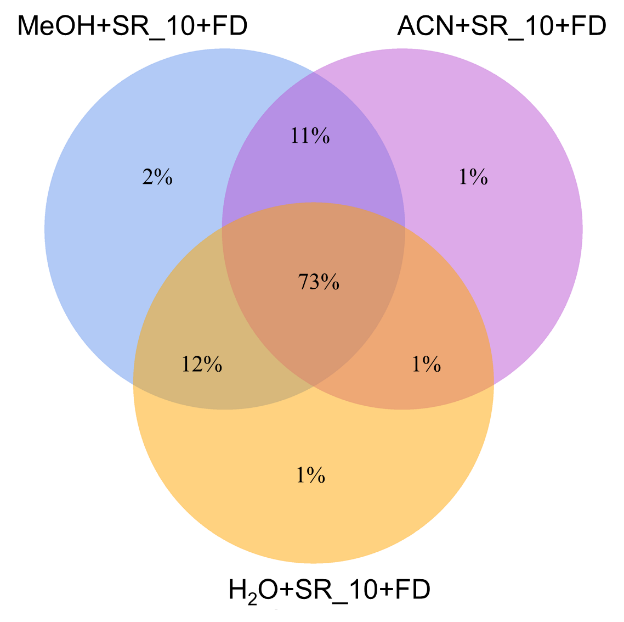  **b**  **a**  **d** |

Fig. S5 Venn diagram representing the distribution of features in fresh (FR, a and b) and freeze-dried (FD, c and d) fecal extracts using MeOH, ACN and H_2_O as extraction solvents at solvent ratio 5 and 10, results in positive electron spray ionization mode.


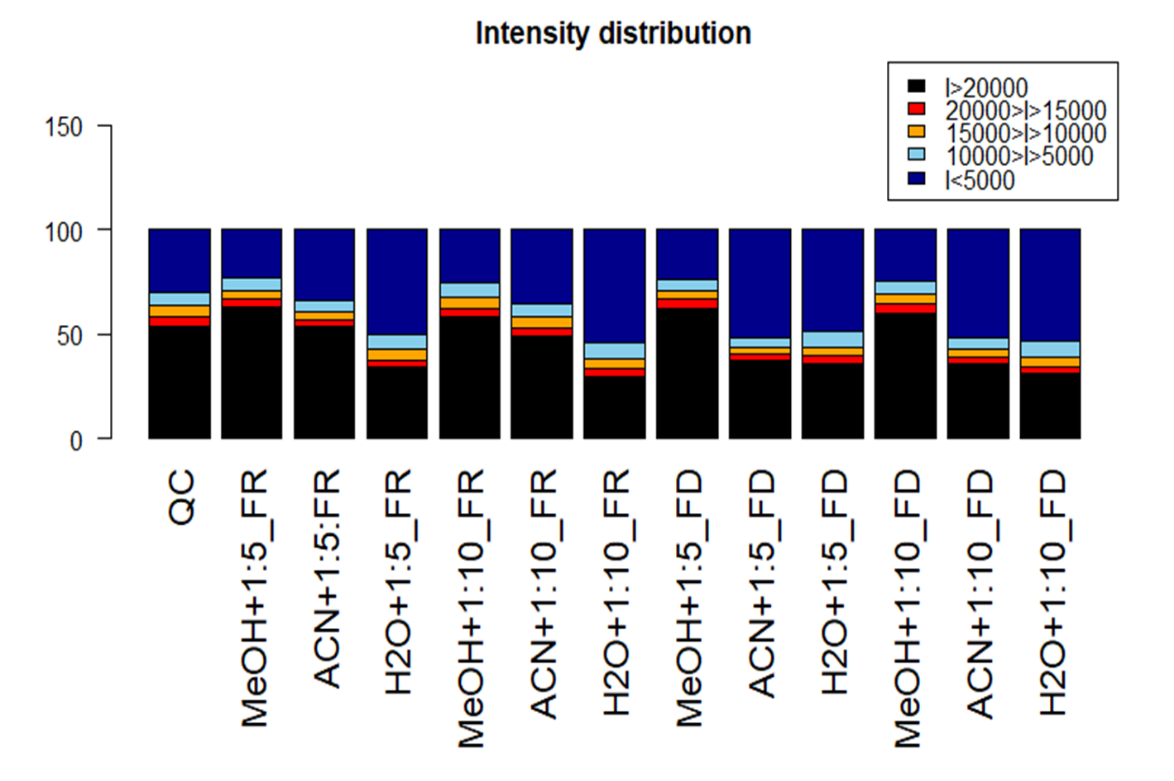


Fig. S6 Distribution of feature intensities in fresh (FR) and freeze-dried (FD) fecal extracts using MeOH as extraction solvents at solvent ratio (SR) 5 and 10, results in positive electron spray ionization mode.

| 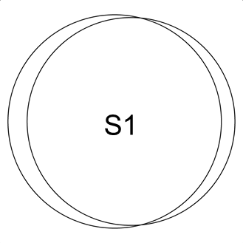 | 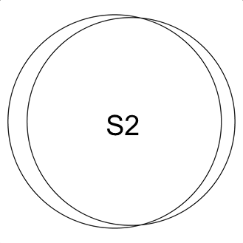 | 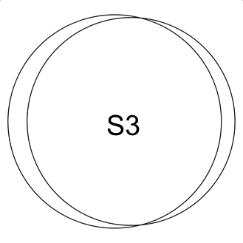 | 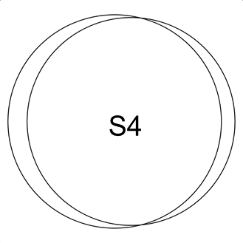 |
| --- | --- | --- | --- |
| 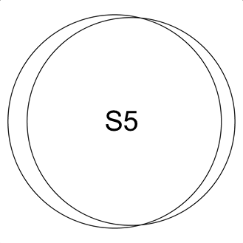 | 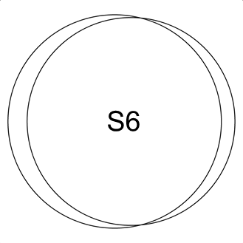 | 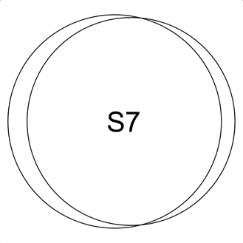 | 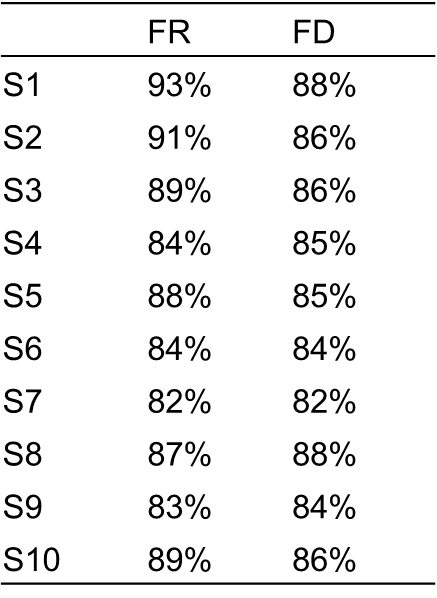 |
| 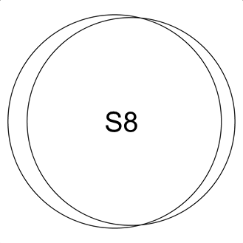 | 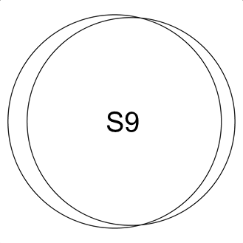 | 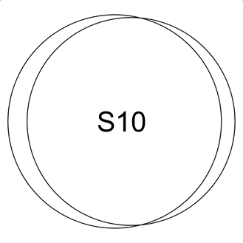 |  |

Fig. S7 Venn diagram representing the distribution of features in fresh (FR, left circle) and freeze-dried (FD, right circle) fecal extracts of ten individuals, results in positive electron spray ionization mode.

| 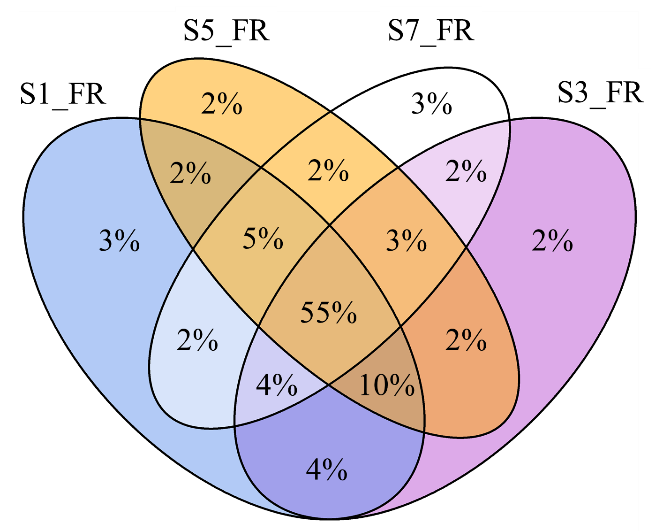  **a** |  | 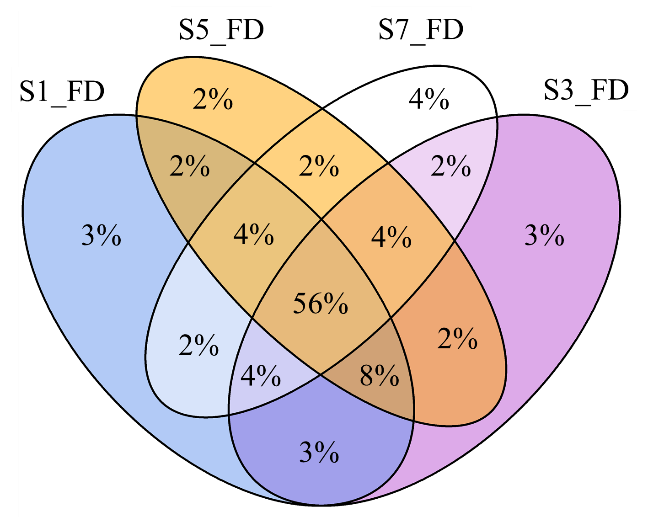  **b** |
| --- | --- | --- |

Fig. S8 Venn diagram representing the distribution of feature in fresh (FR, a) and freeze-dried (FD, b) fecal extracts from four random individuals (S1, S3, S5, S7), results in positive electron spray ionization mode.

| 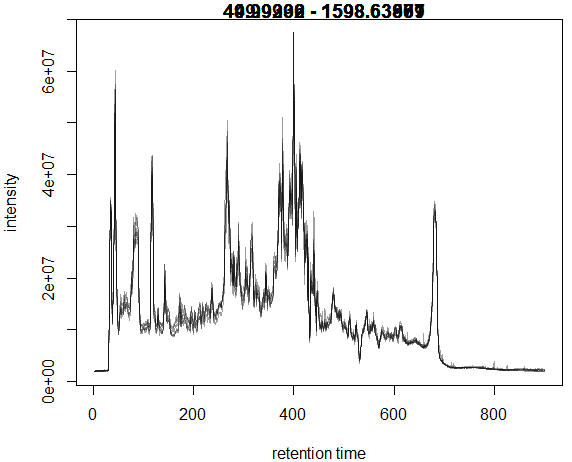 | 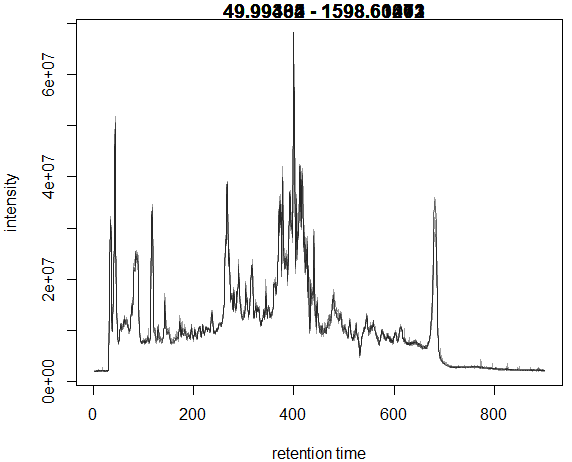 |
| --- | --- |

Fig. S9 Total ion chromatogram of filtered (left) and non-filtered (right) fecal samples (n=5).

| 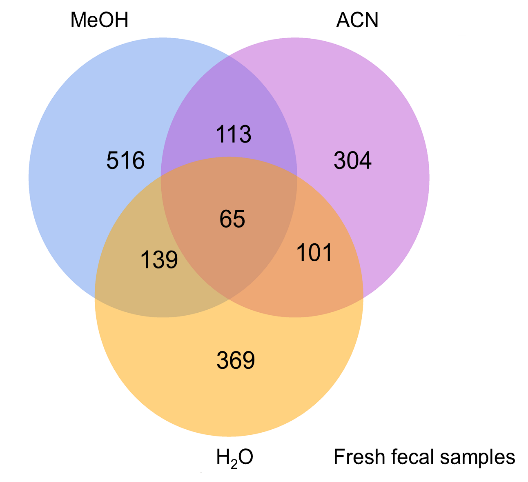 | 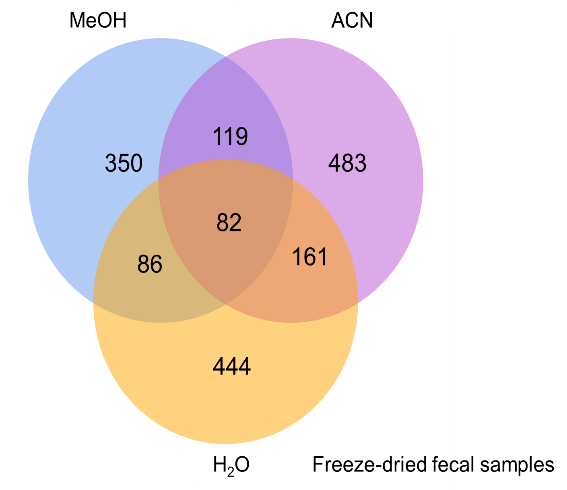 | 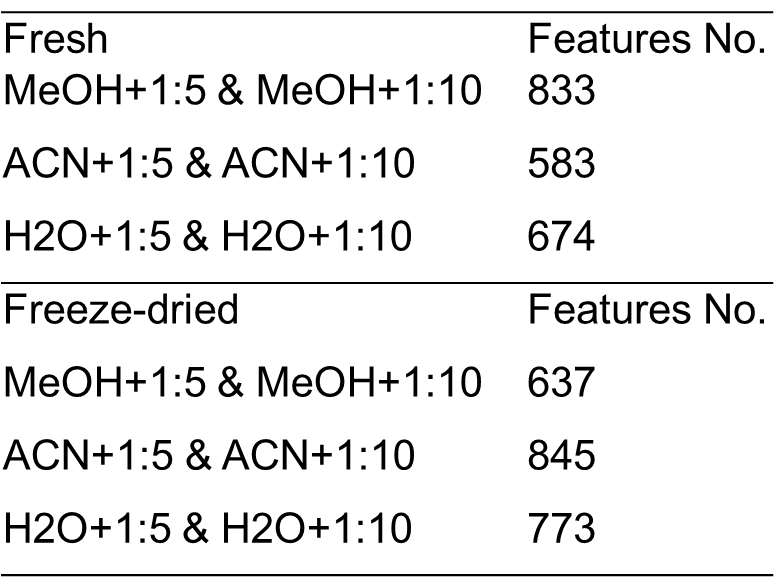 |
| --- | --- | --- |

Fig. S10 Features with stable intensities regardless of dilution (SR5 and SR10) assessed by CV<30%. These features may reflect e.g. contaminants, instrument artefacts or actual features outside of the linear range.
